# Characterization of an APP/tau rat model of Alzheimer’s disease by positron emission tomography and immunofluorescent labeling

**DOI:** 10.1186/s13195-021-00916-2

**Published:** 2021-10-16

**Authors:** Thomas Filip, Severin Mairinger, Joerg Neddens, Michael Sauberer, Stefanie Flunkert, Johann Stanek, Thomas Wanek, Nobuyuki Okamura, Oliver Langer, Birgit Hutter-Paier, Claudia Kuntner

**Affiliations:** 1 Preclinical Molecular Imaging, AIT Austrian Institute of Technology GmbH, 2444 Seibersdorf, Austria; 2 Department of Biomedical Research, Medical University Vienna, Vienna, Austria; 3 Department of Clinical Pharmacology, Medical University of Vienna, Vienna, Austria; 4 Neuropharmacology, QPS Austria GmbH, Grambach, Austria; 5 Department of Biomedical Imaging and Image-guided Therapy, Medical University of Vienna, Vienna, Austria; 6 Division of Pharmacology, Faculty of Medicine, Tohoku Medical and Pharmaceutical University, Sendai, Japan

**Keywords:** Alzheimer’s disease, Immunohistochemistry, Rat model, [^11^C]PiB, [^18^F]THK-5317

## Abstract

**Background:**

To better understand the etiology and pathomechanisms of Alzheimer’s disease, several transgenic animal models that overexpress human tau or human amyloid-beta (Aβ) have been developed. In the present study, we generated a novel transgenic rat model by cross-breeding amyloid precursor protein (APP) rats with tau rats. We characterized this model by performing positron emission tomography scans combined with immunofluorescent labeling and cerebrospinal fluid analyses.

**Methods:**

APP/Tau rats were generated by cross-breeding male McGill-R-Thy1-APP transgenic rats with female hTau-40/P301L transgenic rats. APP/Tau double transgenic rats and non-transgenic (ntg) littermates aged 7, 13, and 21 months were subjected to dynamic [^11^C] PiB scan and dynamic [^18^F]THK-5317 scans. For regional brain analysis, a template was generated from anatomical MR images of selected animals, which was co-registered with the PET images. Regional analysis was performed by application of the simplified reference tissue model ([^11^C]PiB data), whereas [^18^F]THK-5317 data were analyzed using a 2-tissue compartment model and Logan graphical analysis. In addition, immunofluorescent labeling (tau, amyloid) and cerebrospinal fluid analyses were performed.

**Results:**

[^11^C]PiB binding potential (*BP*_*ND*_) and [^18^F]THK-5317 volume of distribution (*V*_*T*_) showed an increase with age in several brain regions in the APP/Tau group but not in the ntg control group. Immunohistochemical analysis of brain slices of PET-scanned animals revealed a positive correlation between Aβ labeling and [^11^C]PiB regional *BP*_*ND*_. Tau staining yielded a trend towards higher levels in the cortex and hippocampus of APP/Tau rats compared with ntg littermates, but without reaching statistical significance. No correlation was found between tau immunofluorescence labeling results and the respective [^18^F]THK-5317 *V*_*T*_ values.

**Conclusions:**

We thoroughly characterized a novel APP/Tau rat model using combined PET imaging and immunofluorescence analysis. We observed an age-related increase in [^11^C]PiB and [^18^F]THK-5317 binding in several brain regions in the APP/Tau group but not in the ntg group. Although we were able to reveal a positive correlation between amyloid labeling and [^11^C]PiB regional brain uptake, we observed relatively low human tau and amyloid fibril expression levels and a somewhat unstable brain pathology which questions the utility of this animal model for further studies.

**Supplementary Information:**

The online version contains supplementary material available at 10.1186/s13195-021-00916-2.

## Background

Alzheimer’s disease (AD) is the most common form of dementia in the elderly. It is typically characterized by: (1) neurofibrillary tangles (NFTs) consisting of intracellular aggregates of abnormally phosphorylated tau (p-tau) protein, (2) abundant amyloid plaques, formed by extracellular aggregates of amyloid-beta (Aβ) peptides, (3) chronic neuroinflammation and (4) neuronal injury and loss [1]. The clinical diagnosis of AD relies on neurological testing, medical imaging, and biomarkers in the cerebrospinal fluid (CSF) and blood [2]. From the molecular imaging techniques, positron emission tomography (PET) significantly contributed to this field with the development of the thioflavin T derivative PET radiotracer ^11^C-labeled Pittsburgh Compound-B ([^11^C]PiB) for imaging Aβ [3, 4]. In addition, a series of tau-selective PET radiotracers were developed, namely the PBB family (e.g. [^11^C]PBB3) [5], the T80x family (e.g. [^18^F]T807) [6] and the THK family (e.g. [^18^F]THK-5117) [7, 8]. However, even with these advanced diagnostic methods at hand, the coexistence of multiple pathological characteristics in AD patients makes it difficult to clarify the mechanisms of etiology.

To overcome the blurring effect of multiple pathologies and better understand AD’s diverse pathogenic mechanisms, several transgenic animal models that overexpress human tau or human Aβ have been developed [9–12]. Historically, mice were preferred over rats for transgenesis mainly due to technical reasons [13]. However, for neuroimaging, rats offer numerous advantages over mice, such as a larger body and brain size leading to a lower occurrence of the partial volume effect and facilitating multiple blood or CSF sampling [14]. Different transgenic rat models for AD were developed in the last years, such as the McGill-R-Thy1-APP rat [15] or the TgF344-AD rat [16]. Examples for tau-specific models are the SHR72, SHR24, and hTau-40/P301L rats [17–19].

Many transgenic animal neurons express excessive amounts of the target protein. Well-known and frequent problems of this approach include accumulation of protein due to unphysiologically strong expression, affecting the function and viability of cells simply by overwhelming the biochemical machinery rather than by target protein-specific effects. Due to these reasons the weak transgenic tau model (hTau-40/P301L rats) described by Kohonen et al. [18] raised our interest in trying to exacerbate pathology by crossing the rats with an APP transgenic line. This approach was already suggested in the publication by Kohonen et al.. Such exacerbating effects of crossing different disease models were shown to occur in mice [20]. APP mice were crossed with tau mice (expressing wild-type human tau without pathogenic mutation), resulting in a double-transgenic strain that developed both plaques and NFTs. While the presence of tau did not affect amyloid pathology, the presence of amyloid greatly enhanced NFT formation in several brain regions of these double-transgenic mice.

In the present study, we translated this strategy to rats by crossing APP rats with hTau rats to generate APP/Tau rats. PET scans were performed in female and male APP/Tau rats and non-transgenic littermates aged 7, 13, and 21 months using [^11^C]PiB and [^18^F]THK-5317 (corresponding to the (*S*)-enantiomer of racemic [^18^F]THK-5117) to test the suitability of the newly developed model for monitoring of disease progression utilizing in vivo imaging. In addition, brain and CSF were analyzed for the presence of amyloid and human recombinant tau by immunofluorescent labeling and immunosorbent assay to provide a holistic assessment of this new model.

## Materials and methods

### Chemicals

All chemicals were purchased from Sigma-Aldrich Handels GmbH (Vienna, Austria) and used without further purification. The radiosynthesis of [^18^F]THK-5317 and [^11^C]PiB was performed following established protocols [8, 21]. [^18^F]THK-5317 (*n* = 31) was synthesized with a decay-corrected radiochemical yield of 13 ± 7% and a radiochemical purity of 93 ± 7% in a total synthesis time of 76 min. [^11^C]PiB (*n* = 21) was synthesized with a decay-corrected radiochemical yield of 6 ± 2% and a radiochemical purity of 99.7 ± 0.6% in a total synthesis time of 26 min. Molar activities and injected masses are summarized in Table 1.

**Table 1:** Overview of group size (n), age, weight, injected activity, injected mass, and molar activity for [^11^C]PiB and [^18^F]THK-5317 PET imaging

[^11^C]PiB	n	Age (months)	Weight (g)	Inj. Activity (MBq)	Inj. Mass (nmol/kg)	Molar activity (GBq/μmol)
Female APP/hTau	3	6.7 ± 0.3	257.7 ± 32.7	23.6 ± 6.1	3.2 ± 1.2	29.9 ± 5.0
Male APP/hTau	3	7.1 ± 0.1	493.0 ± 51.4	26.3 ± 0.7	6.1 ± 4.1	16.2 ± 16.8
Female APP/hTau	8	12.1 ± 0.6	334.5 ± 30.8	14.8 ± 2.2	0.4 ± 0.1	115.1 ± 12.9
Male APP/hTau	10	12.4 ± 0.7	540.6 ± 47.6	16.7 ± 3.3	0.3 ± 0.0	109.3 ± 22.7
Female APP/hTau	8	20.5 ± 0.5	347.0 ± 48.8	21.1 ± 1.1	0.6 ± 0.2	21.1 ± 1.1
Male APP/hTau	10	20.6 ± 0.3	606.9 ± 43.8	31.0 ± 16.8	0.5 ± 0.3	31.0 ± 16.8
**[**^**18**^**F]THK-5317**	**n**	**Age (months)**	**Weight (g)**	**Inj. Activity (MBq)**	**Inj. Mass (nmol/kg)**	**Molar activity (GBq/μmol)**
Female ntg	6	7.0 ± 0.2	273.3 ± 17.6	19.2 ± 5.2	1.5 ± 0.6	52.5 ± 16.7
Female APP/hTau	7	7.0 ± 0.4	263.4 ± 21.2	17.8 ± 3.1	1.4 ± 1.7	149.9 ± 122.8
Male ntg	6	7.1 ± 0.1	491.2 ± 57.5	16.2 ± 3.8	0.7 ± 0.1	53.7 ± 15.2
Male APP/hTau	7	7.1 ± 0.3	485.6 ± 65.8	17.4 ± 3.8	0.3 ± 0.1	182.0 ± 118.6
Female ntg	6	12.8 ± 0.5	300.3 ± 38.0	15.0 ± 1.5	0.9 ± 0.5	71.3 ± 45.1
female APP/hTau	11	12.5 ± 0.5	319.7 ± 28.7	11.6 ± 4.3	0.7 ± 0.6	83.6 ± 66.3
Male ntg	2	13.4 ± 0.4	632.5 ± 0.7	8.8 ± 3.9	0.4 ± 0.5	194.8 ± 257.6
male APP/hTau	10	12.9 ± 0.4	520.3 ± 53.4	15.9 ± 4.3	0.4 ± 0.3	135.4 ± 99.1
Female ntg	4	20.7 ± 0.4	354.0 ± 82.9	18.5 ± 2.6	0.9 ± 1.0	103.1 ± 46.1
Female APP/hTau	6	20.7 ± 0.6	342.8 ± 52.1	16.2 ± 1.2	0.2 ± 0.1	388.9 ± 236.8
Male ntg	4	21.0 ± 0.3	538.3 ± 54.4	16.3 ± 2.5	0.6 ± 0.5	88.0 ± 70.8
Male APP/hTau	10	20.9 ± 0.3	594.5 ± 48.9	15.2 ± 2.6	0.2 ± 0.3	181.4 ± 112.6

### Breeding of APP/tau rats

APP/Tau rats were developed by cross-breeding male McGill-R-Thy1-APP transgenic rats with Swedish double mutation (K670N/M671L) and Indiana mutation (V717F) [15] with female hTau-40/P301L transgenic rats [18]. Genotyping was performed twice by collecting the tail tips of not more than 1 mm length from each animal, once before the start of the study and the second time during tissue sampling. Genomic DNA was prepared from tail biopsy by alkaline lysis and subsequent neutralization of the lysate. Real-time polymerase chain reactions (qPCR) using specific primers that recognize human tau (Fwd: 5′-TGG TCC GTA CTC CAC CCA AG − 3′; Rev.: 5′- GAG GTC ACC TTG CTC AGG TCA A-3′) or human APP (Fwd: 5′-CGG AGC AGA CAC AGA CTA TGC − 3′, Rev.: 5′- CCA TCC TCA TCG TCC TCG T-3′) were used to distinguish double transgenic, single transgenic and non-transgenic animals (CFX Connect Real-Time PCR Detection System (Bio-Rad, Hercules, California, USA)). Animals were bred and aged in groups separated by sex in the AAALAC-accredited animal facility of QPS Austria GmbH and transferred to the AIT animal facility before the start of the experiments.

### Animals used in experiments

Adult female and male APP/Tau double transgenic rats and non-transgenic (ntg) littermates aged between 7 and 21 months on a Wistar background were used for the study. The sample size was calculated by power analysis (G*Power 3.1, University Kiel, Germany) [22], assuming a meaningful effect size of 1.8, a standard deviation of 0.5, an α error of 0.05, and power of 80% yielding *n* = 6 per group. In some groups, the sample size was not completely achieved due to losses during the experiment. Exclusion criteria were abnormal behavior, signs of sickness, more than a 15% reduction in body weight, and death. A summary of study groups, number of animals, sex, age, and injected radioactivity amount is given in Table 1. At AIT, animals were housed in the same groups as at QPS Austria GmbH. At both locations, animals were housed in a temperature- and humidity-controlled facility under a cycle of 12 h of light and 12 h of dark, with free access to standard laboratory animal diet (ssniff R/M-H, ssniff Spezialdiäten GmbH, Soest, Germany) and water ad libitum. An acclimatization period of at least one week after transfer to AIT was allowed before animals were used in the experiments. Selected animals (*n* = 1–2 per group, 12 male and 11 female in total) underwent surgery for cannulation of the femoral artery and vein shortly before the [^18^F]THK-5317-PET-scan to obtain an arterial input function (IF) as described previously [23].

Apart from the 79 rats used in the imaging experiments, the study comprised 33 additional rats, which were used to practice the surgical procedure before each experimental part of the study and as spare animals due to losses during the imaging session (e.g. technical problems with scanner, deceased during scanning or deceased during surgery).

The study was approved by the national authorities (notification number: LF1-TVG-48/033–2017; Amt der Niederösterreichischen Landesregierung, Austria), and study procedures were following the European Communities Council Directive of September 22, 2010 (2010/63/EU). The experimental animal data reported in this study comply with the ARRIVE (Animal Research: Reporting of in Vivo Experiments) guidelines 2.0 [24].

### PET imaging

For PET imaging, a small animal PET scanner (Focus 220™, Siemens Healthineers) was used, offering a 7.6 cm axial and a 19 cm transaxial field-of-view allowing simultaneous scanning of two rats in a custom-made dual imaging cradle (side per side) [25]. Before PET imaging, anesthesia was induced in a dedicated induction box with isoflurane mixed with air as carrier gas. Afterward, the animals were positioned on the dual imaging cradle with continuing anesthesia. Then the lateral tail vein was catheterized after warming of the tail. Animals were warmed throughout the experiment, and body temperature and respiratory rate were constantly monitored. All animals underwent a 60-min dynamic [^18^F]THK-5317 scan. Transgenic animals additionally underwent a 60-min dynamic [^11^C]PiB scan before the [^18^F]THK-5317 scan. The time delay between the [^11^C]PiB scan and the [^18^F]THK-5317 scan was between 7 and 16 days (mean 10 ± 3 days) to allow sufficient recovery of animals. PET scanning was initiated at the start of radiotracer injection (slow bolus over ~ 40 s), and list-mode data were acquired with an energy window of 350–750 keV and a 6 ns timing window. A 10-min transmission scan was performed using a rotating ^57^Co-point source before each PET scan for attenuation correction. After the [^18^F]THK-5317 scan, a blood sample was collected into a small tube (Microvette CB 300 LH, Sarstedt AG & Co, Nümbrecht, Germany) by puncture of the retrobulbar plexus under anesthesia. Then animals were euthanized by intravenous injection of pentobarbital (Eutasol 40% ad us vet, Virbac GmbH, Austria), cerebrospinal fluid was sampled (13 and 21 months old rats only), and transcardial perfusion using saline was performed. After that, the brain was removed, divided into hemispheres, fixed in 4% paraformaldehyde (PFA) in phosphate-buffered saline (PBS) for 2 h at room temperature, and equilibrated in 15% saccharose/PBS overnight. After washing with PBS, brains were embedded in a peel-a-way form in Tissue-Tek (Sakura Finetek Germany GmbH) to allow sagittal cutting, snap-frozen using isopentane and liquid nitrogen, and stored at − 80 °C.

### Ex vivo analysis of samples

Blood was centrifuged to obtain plasma (17,000 g, 4 °C, 1 min), and radioactivity concentrations in the brain, CSF, blood, and plasma samples were measured in a gamma-counter (HIDEX AMG Automatic Gamma Counter, Turku, Finland). Empty and full tubes were weighted to obtain sample weight. The gamma-counter was calibrated using a series of tubes with decreasing activity of an ^18^F- and ^11^C-solution. Data from the gamma-counter expressed in kBq/g were decay-corrected to the time of radiotracer injection. Then, data were corrected by the injected activity and bodyweight of the animals and expressed as standardized uptake value (SUV).

### Magnetic resonance imaging

To generate a template for regional brain analysis, selected animals (*n* = 1–2 / group) underwent anatomical magnetic resonance (MR) imaging using a 1 T scanner (ICON, Bruker). Animals were anesthetized using isoflurane and positioned prone in the imaging cradle equipped with the rat head coil. As the male 21-month old rats did not fit onto the animal cradle, MR images from the head were measured post mortem. MR images were acquired using T1-IG-FLASH-3D sequences in three different orientations (coronal, sagittal, axial). For the axial orientation the parameters were as follows: TE: 6.2 ms, TR: 24.5 ms, averages: 1, FOV: 35 × 35 × 42 mm^3^, matrix: 140 × 140 × 56, resolution: 0.25 × 0.25 × 0.75 mm/voxel, scan time: 4 min, flip angle: 30°.

### PET image data analysis

Dynamic list-mode data from the 60-min scans were sorted into three-dimensional sinograms according to the following frame sequence: 8 × 5 s, 2 × 10 s, 2 × 30 s, 3 × 60 s, 2 × 150 s, 2 × 300 s, and 4 × 600 s. PET images were reconstructed by Fourier rebinning of the 3D sinograms followed by two-dimensional filtered back projection with a ramp filter resulting in a voxel size of 0.6 × 0.6 × 0.8 mm^3^. The standard data correction protocol, including normalization, attenuation, and decay correction, was applied to the data. Before each measurement series, the PET scanner was cross-calibrated with the activimeter by imaging a phantom with a known activity concentration of an ^18^F- and ^11^C-radiotracer solution. MR images from one group were normalized in size using the PMOD fusion tool (PFUS version 4.002, PMOD Technologies Ltd., Zurich, Switzerland). Then, the Schiffer rat brain atlas implemented in PMOD was co-registered to the normalized MR image. To simplify analysis and avoid partial volume effects, smaller brain regions were merged to yield 9 larger brain regions: frontal cortex, somatosensory cortex, cortex, hippocampus, thalamus, striatum, hypothalamus, cerebellum, and brainstem. Moreover, all cortical regions were merged, yielding the ‘cortex all’ region. After that, the normalized MR was co-registered with the PET image by rigid transformation, and the individual time-activity curves (TACs) expressed in kBq/cc were extracted. This procedure was performed for all [^11^C]PiB and [^18^F]THK-5317 scans. The operator performing data analysis and kinetic modeling was aware of group assignments. Since a standardized procedure (definition of PET brain regions, see above) was used for quantitative analysis, an influence on data outcome was not expected.

### Kinetic modeling

Kinetic modeling was performed using the kinetic model tool implemented in PMOD. [^11^C]PiB images were analyzed by using the simplified reference tissue model (SRTM) and the Logan reference tissue model (LRT) with the cerebellum as a reference region to obtain the binding potential (*BP*_*ND*_) from all analyzed brain regions. Transgenic animals were analyzed at 7 months (baseline time point), 13 months, and 21 months.

For the [^18^F]THK-5317 data, two different methods for kinetic modeling were applied. From the blood-based models, a one-tissue two-rate constant compartmental model (1T2K) and a two-tissue four-rate constant compartmental model (2T4K) were fitted to the brain data. The goodness of fits was assessed based on the Akaike information criterion (AIC) values. The vascular volume fraction in the rat brain was fixed at 0.05. Logan graphical analysis was applied to the [^18^F]THK-5317 PET data to obtain a model-independent estimate of the volume of distribution (*V*_*T*_). For the blood-based models (1T2K, 2T4K, Logan graphical analysis), a sex-specific metabolite corrected population-based plasma and blood IF was used for modeling male and female rats [23]. The IF was constructed by linear interpolation of the measured arterial blood activity data and by multiplication with the ratio of plasma to whole blood activity. Moreover, the radiometabolite data for the 5-, 20-, and 60-min time points were used to generate a metabolite curve by fitting a monoexponential decay function to the fraction of unmetabolized radiotracer versus time. This curve was multiplied with the plasma activity data to obtain a metabolite-corrected arterial IF. In individual animals, the population-based sex-specific IFs were adjusted based on the injected radioactivity and the bodyweight as described before [23] to obtain the IF in units of kBq/mL needed for compartmental modeling. The correlation between *V*_*T*_*s* based on individual IF and population-based IF was evaluated to validate the population-based IF approach. In addition, [^18^F]THK-5317 PET data were analyzed with the SRTM and the LRT model using the cerebellum as a reference region [26].

### Immunofluorescence and CSF analysis

Brains were sagittally sectioned at 10 μm thickness on a cryotome (CM3050S, Leica). Sections of transgenic and non-transgenic 7-, 13- and 21-month old animals were labeled with the following primary antibodies: mouse anti-human tau monoclonal antibody (TAU-13; BioLegend, 835,201), rabbit anti-Abeta fibrils polyclonal antibody (LOC; Merck Millipore, AB2287), goat anti-GFAP polyclonal antibody (abcam, ab53554), and guinea pig anti-IBA1 polyclonal antibody (Synaptic Systems, 234,004). Primary antibodies were detected with the following secondary antibodies raised in donkey: Anti-mouse conjugated with DyLight 650 (Thermo Fisher, SA5–10169), anti-rabbit conjugated with DyLight 755 (Thermo Fisher, SA5–10043), anti-sheep conjugated with Alexa Fluor 488 (abcam, ab150177), and anti-guinea pig conjugated with Cy3 (Jackson ImmunoResearch, 706–165-148). Cell nuclei were stained with DAPI. Whole slide scans of all five fluorescence channels were obtained with a Zeiss AxioScan Z1. Images were quantitatively analyzed for the immunoreactive area (IR area) in the cortex and hippocampus using Image Pro 10 software (Media Cybernetics).

The amount of Aβ1–38, Aβ1–40, Aβ1–42, and total tau in the cerebrospinal fluid (CSF) of 13- and 21-month old animals was evaluated by immunosorbent assay (V-PLEX Plus Aβ Peptide Panel 1 (6E10) Kit and V-PLEX Human Total Tau Kit; Meso Scale Diagnostics, LLC). The CSF was diluted 1:20 in the provided diluent and added according to the manufacturer’s protocol.

### Statistics

Statistical testing was performed using GraphPad Prism 9.1.0 software (GraphPad Software, La Jolla, CA, USA). Differences between transgenic groups, sex, and age were analyzed by one-way ANOVA followed by Tukey’s multiple comparison test. Differences between immunofluorescent labeling results (IR area) in the selected age groups were analyzed by a two-tailed unpaired t-test. Correlations were assessed by calculating Pearson’s correlation coefficient (*r*). The level of statistical significance was set to *p* < 0.05. All values are given as mean ± standard deviation (SD) unless stated otherwise.

## Results

### Quantification of human amyloid fibrils, tau protein, and Aβ in APP/tau rats

**Fig. 1:**
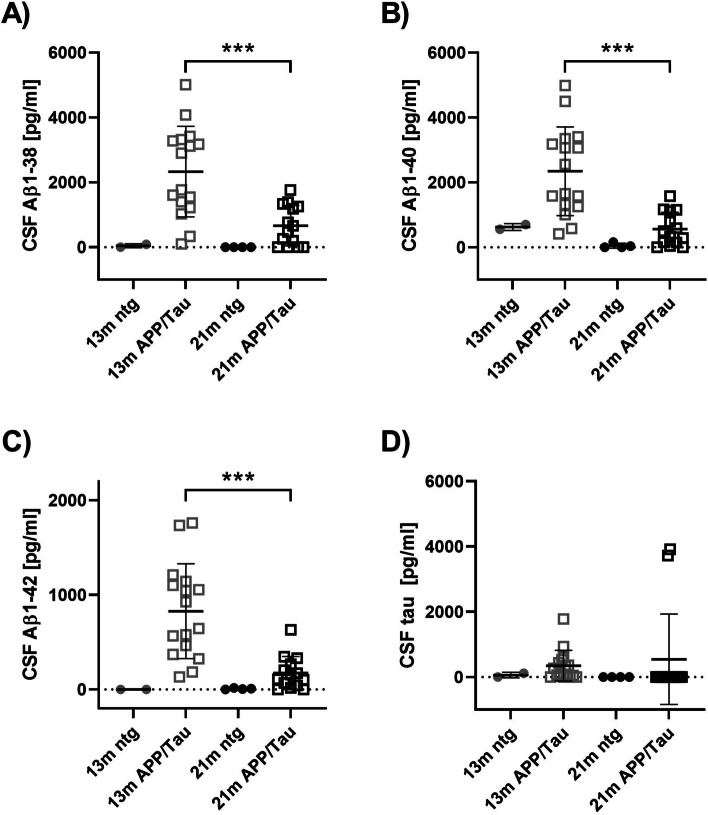
Quantification of human Aβ1–38 (**A**), Aβ1–40 (**B**), Aβ1–42 (**C**) and tau (**D**) in the CSF of 13-month and 21-month old rats (13 months: APP/Tau: *n* = 16, ntg: *n* = 2; 21 months: APP/Tau: *n* = 14, ntg: *n* = 4). Bars represent mean ± SD; ***p < 0.001, one-way ANOVA followed by Tukey’s multiple comparison test

**Fig. 2:**
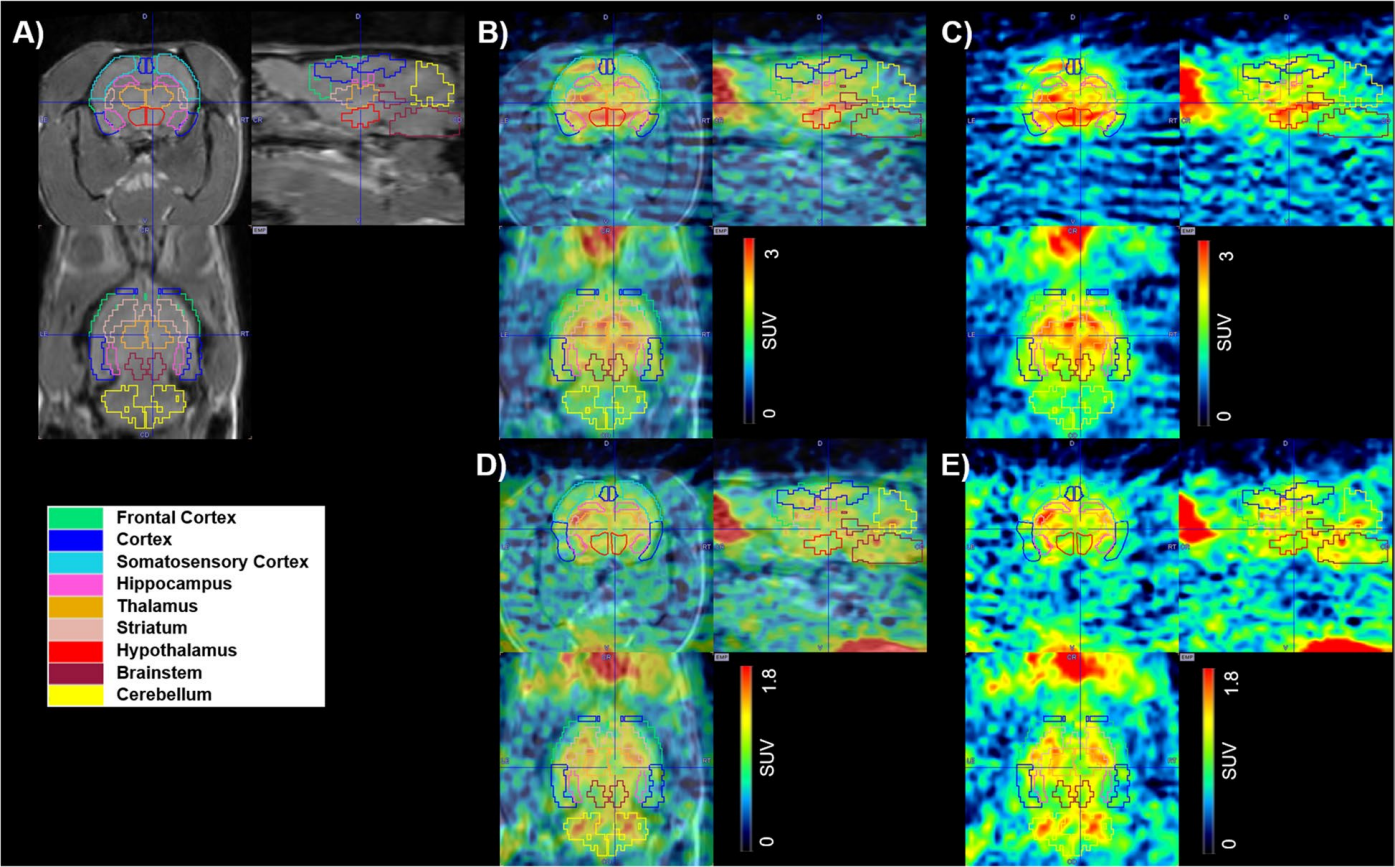
Representative MR (**A**), co-registered PET/MR (**B**, **D**) and PET summation images (30–60 min p.i.) (**C**, **E**) of one male 21 months old APP/Tau rat (600 g) for [^11^C]PiB (60.7 MBq) (**B**, **C**) and [^18^F]THK-5317 (20.2 MBq) (**D**, **E**), respectively. Radioactivity is expressed in units of SUV. The outlined regions of interest are shown: frontal cortex (light green), cortex (blue), somatosensory cortex (light blue), hippocampus (pink), thalamus (orange), striatum (nude), hypothalamus (red), brainstem (brown) and cerebellum (yellow)

**Fig. 3:**
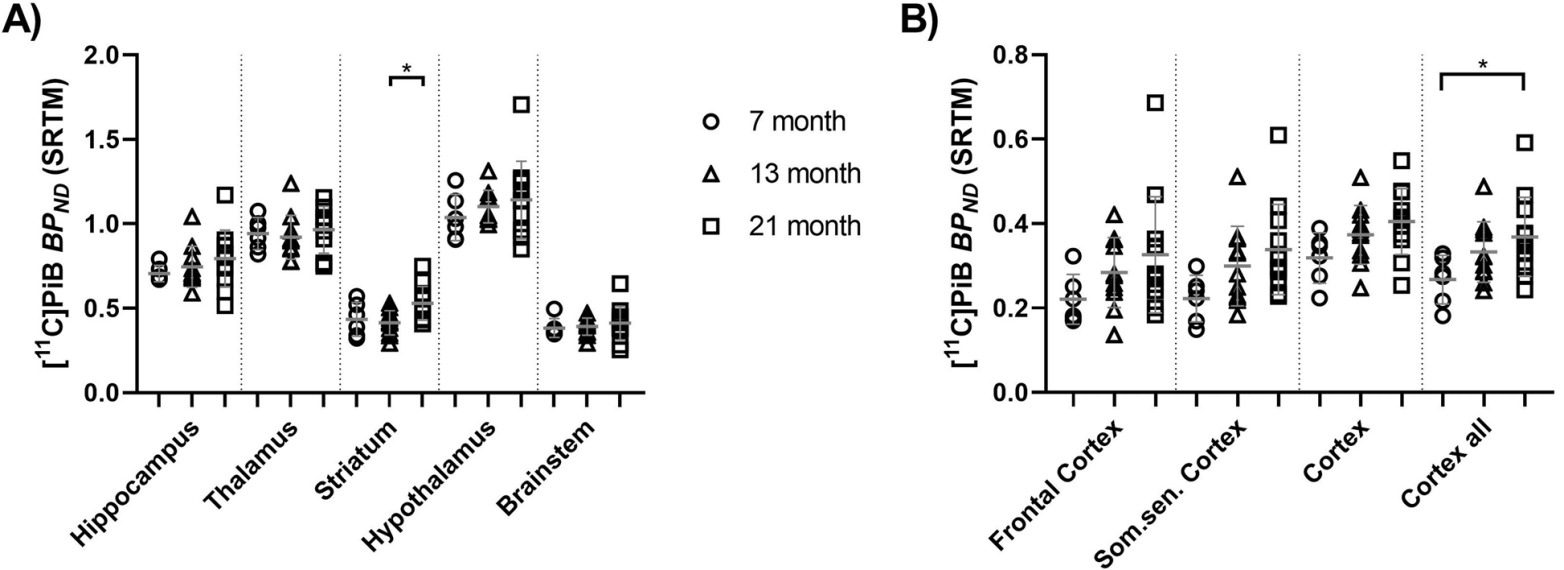
[^11^C]PiB binding potential (*BP*_*ND*_) obtained with the simplified reference tissue model (SRTM) in central (**A**) and cortical (**B**) brain regions of APP/Tau rats pertaining to different age groups (circles: 7 months, *n* = 6; triangles: 13 months, *n* = 11; squares: 21 months, *n* = 12). Male and female animals were grouped, and only amyloid-positive animals based on immunofluorescent labeling analysis were included. Som.sen. Cortex = somatosensory cortex. * *p* < 0.05, one-way ANOVA followed by Tukey’s multiple comparisons test

Results from immunofluorescent labeling are shown in the Supplementary materials Figure 1S (7 months), Figure2S (13 months), and Figure 3S (21 months). Tau labeling in the 21-month old rats was performed using a different Tau13 antibody batch leading to overall lower IR area values. At the age of 7 months, anti-amyloid fibril labeling was similar between ntg and APP/Tau rats, while significantly higher tau labeling was observed in the cortex and hippocampus of APP/Tau rats compared to ntg littermates (cortex *p* = 0.004; hippocampus *p* = 0.044, Fig. 1SB + D). At the age of 13 months, there was an increase in human Aβ and tau in the cortex and hippocampus of APP/Tau rats relative to ntg littermates. However, this increase did not reach statistical significance due to the low number of analyzed brain slices in the ntg group (*n* = 2; Fig. 2S). At 21 months, significantly higher Aβ staining was observed in the cortex and hippocampus of APP/Tau rats compared to ntg littermates (cortex *p* = 0.049; hippocampus *p* = 0.006, Fig. 3S). In addition, there was a trend towards higher tau staining in the cortex and hippocampus of APP/Tau rats relative to ntg littermates, but statistical significance was not reached. Interestingly, several APP/Tau rats showed low or no expression of human tau and Aβ, although genotyping confirmed the presence of both genes (see Supplementary materials Table 1S). No differences in the IR areas of GFAP and IBA1 to label astrocytosis and activated microglia, respectively, were found between APP/Tau rats and ntg littermates at the age of 13 and 21 months (Fig. 2S, 3S).

Analysis of CSF samples revealed significantly higher Aβ1–38, Aβ1–40, Aβ1–42 levels in 13-month old APP/Tau compared to 21-months old APP/Tau animals (Fig. 1). At 13 months, CSF Aβ levels were higher in APP/Tau rats than ntg littermates. However, this difference did not reach significance due to the low number of analyzed CSF samples in the ntg group (*n* = 2, Fig. 1). Human tau levels in CSF samples of 13-month old APP/Tau rats were shallow, while only two CSF samples of 21-month old APP/Tau rats were tau positive.

### [^11^C]PiB PET scans and correlation with immunofluorescent labeling

All ntg and APP/Tau rats underwent a [^18^F]THK-5317-PET scan, whereas [^11^C]PiB-PET scans were only performed in the APP/Tau group. Exemplary PET and MR images of one male 21-month old APP/Tau rat are shown in Fig. 2.

For the [^11^C]PiB scans, *BP*_*ND*_ was obtained as an outcome parameter by using the SRTM and the LRT model with the cerebellum as a reference region (see Supplementary materials Table 2S). As no significant effect of sex on *BP*_*ND*_ in the studied brain regions was found, male and female animals were grouped. There was a good correlation between *BP*_*ND*_ values obtained with LRT and SRTM analysis (*r* = 0.9753, *p* < 0.0001, see Supplementary materials Figure 4S). The somatosensory cortex, cortex (all cortical regions except frontal and somatosensory cortex), cortex all (all cortical regions), and striatum showed a trend towards an age-dependent increase in *BP*_*ND*_. Immunofluorescent labeling showed that some animals did not express human Aβ in the brain with an IR area close to 0. Exclusion of these animals from the statistical analysis revealed significantly higher [^11^C]PiB *BP*_*ND*_ values in the ‘cortex all’ region of 21-month old compared to 7-months old APP/Tau rats (*p* = 0.044) and in the striatum of 21-month old compared to 13-month old APP/Tau rats (*p* = 0.013; see Fig. 3).

**Fig. 4:**
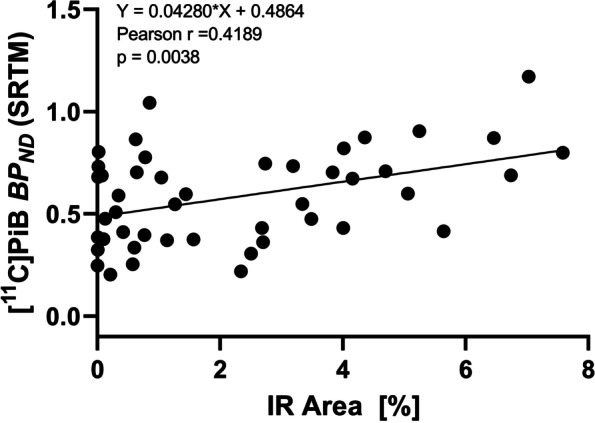
Correlation of [^11^C]PiB *BP*_*ND*_ obtained with simplified reference tissue modeling (SRTM) in the cortex and hippocampus of male and female APP/Tau rats with the corresponding amyloid immunoreactive (IR) area of immunofluorescent labeling (all age groups, *n* = 46; only amyloid-positive animals are included)

Finally, cortical and hippocampal [^11^C]PiB *BP*_*ND*_ (SRTM) showed a weak but significant positive correlation with the respective IR area measured with immunofluorescent labeling (*r* = 0.4189, *p* = 0.0038; Fig. 4).

### [^11^C]PiB PET scans and correlation with CSF markers

There was no correlation of Aβ1–38, Aβ1–40, Aβ1–42 in the CSF with cortical [^11^C]PiB *BP*_*ND*_ values, while a weak but significant positive correlation of CSF tau with cortical *BP*_*ND*_ values was obtained (*r* = 0.5844, *p* = 0.046, see Supplementary materials Figure 5S).

### [^18^F]THK-5317 PET scans and correlation with immunofluorescent labeling

Biodistribution data of [^18^F]THK-5317 in CSF, brain, blood, and plasma obtained from female and male ntg and APP/Tau rats at 60 min after radiotracer injection are summarized in the see Supplementary materials Table 3S. No significant differences were observed between groups.

After testing different kinetic models in the 21-months old rats, the 2T4K compartmental model and Logan graphical analysis were selected as the preferred kinetic models for [^18^F]THK-5317 analysis. Details of the evaluation process to select these models can be found in the Supplementary materials.

**Fig. 5:**
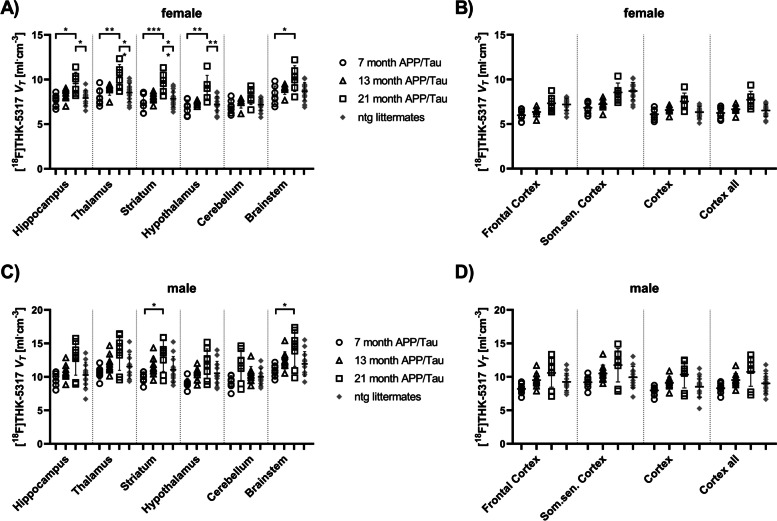
Volume of distribution (*V*_*T*_) of [^18^F]THK-5317 obtained with Logan graphical analysis in 7, 13 and 21 months old female (**A**, **B**) and male (**C**, **D**) APP/Tau and ntg rats (female APP/Tau: 7 months: *n* = 7; 13 months: *n* = 7; 21 months: *n* = 6; female ntg (7–21 months): *n* = 15; male APP/Tau: 7 months: *n* = 7; 13 months: *n* = 10; 21 months: *n* = 8; male ntg (7–21 months): *n* = 12). Som.sen. Cortex = somatosensory cortex. *p < 0.05, **p < 0.01, ***p < 0.001; one-way ANOVA followed by Tukey’s multiple comparisons test

Logan *V*_*T*_s in ntg rats were very similar in the analyzed brain regions for all age groups. Regional *V*_*T*_s for each sex were pooled across all age groups for ntg rats to increase the statistical power. Outcome parameters of kinetic modeling for female and male animals are given in the Supplementary materials, Table 4S and 5S, respectively. In female APP/Tau rats, significantly higher *V*_*T*_s in the hippocampus (*p* = 0.015), thalamus (*p* = 0.003), striatum (*p* = < 0.001), hypothalamus (*p* = 0.003), and brainstem (*p* = 0.04) in 21-month old animals compared to 7-month old animals were found. Moreover, significantly higher *V*_*T*_s in the hippocampus (*p* = 0.03), thalamus (*p* = 0.008), striatum (*p* = 0.005), and hypothalamus (*p* = 0.003) of 21-month old female APP/Tau rats compared to female ntg littermates were found (Fig. 5A). For male APP/Tau rats, significantly higher *V*_*T*_s were obtained in the striatum (*p* = 0.047) and brainstem (*p* = 0.043) of 21-month compared to 7-month old animals (Fig. 5C). However, no significant differences in *V*_*T*_*s* were observed between the 21-month old male APP/Tau rats and male ntg littermates.

Significantly higher *V*_*T*_s in all brain regions of male animals than female animals were observed in ntg and APP/Tau rats (*p* < 0.05, see Table 4S and 5S).

Finally, correlation analysis of cortical and hippocampal [^18^F]THK-5317 *V*_*T*_s with the respective Tau13 IR areas showed no significant correlation between these parameters (see Supplementary materials Figure 8S).

## Discussion

In the present study, a newly developed APP/Tau rat model was characterized utilizing [^11^C]PiB and [^18^F]THK-5317 PET imaging accompanied by immunofluorescence and CSF analysis. The rationale behind selecting [^11^C]PiB and [^18^F]THK-5317 as radiotracers to characterize our APP/Tau rat model was that both radiotracers have been employed before in rat models of AD and have provided an increase in signal with age [27, 28]. In our present study, we applied for the first time total PET quantification by kinetic modeling yielding *BP*_*ND*_ for [^11^C]PiB and *V*_*T*_ for [^18^F]THK-5317 as outcome parameters in male and female APP/Tau and ntg rats at the age of 7, 13 and 21 months, respectively. After PET imaging, brains were extracted and analyzed for human total tau, amyloid, GFAP, and IBA levels.

First, we observed that the newly developed double-transgenic APP/Tau rat model exhibited an unstable brain pathohistology. Although all employed APP/Tau rats were positively genotyped for tau and APP gene expression, the brain pathohistology defined by immunofluorescent labeling did not mirror the genotype with 17% amyloid-negative and 62% tau-negative animals among all 13- and 21-month old APP/Tau rats. Such a discrepancy between genotype and phenotype has also been reported for the hTau-40/P301L rat model, for which 30% of tau transgenic animals did not express human tau protein at detectable levels [18]. Thus, the unstable phenotype in the presented APP/Tau model seems to be inherited from the hTau-40/P301L parent genome.

To overcome this limitation, we excluded the amyloid-negative animals from further analysis of the [^11^C]PiB data. We found significantly higher [^11^C]PiB *BP*_*ND*_ values (+ 38%) in the cortex of 21-month old APP/Tau rats compared to 7-month old animals, which is slightly higher than the differences reported for another AD rat model [28]. Supporting these findings, the histological analysis yielded significantly increasing amyloid levels in cortical and hippocampal regions of APP/Tau rats with age. We obtained a positive correlation between immunofluorescent amyloid labeling and [^11^C]PiB *BP*_*ND*_, which supported the feasibility to follow Aβ-related disease progression in this rat model by PET imaging. Parent et al. [27] performed a longitudinal study in McGill-RThy1-APP transgenic rats using the Aβ radiotracer [^18^F]NAV4694-PET combined with MRI, fMRI, and spatial memory tasks at the age of 9–11 months and 16–19 months. The authors reported on an increase in *BP*_*ND*_ and a decrease of Aβ CSF markers with age in McGill-RThy1-APP transgenic rats, which is in good agreement with findings from our present study.

Approximately 1 week after the [^11^C]PiB-PET scans, APP/Tau and ntg littermates underwent a [^18^F]THK-5317 PET scan. In both female and male APP/Tau rats, we observed a significant increase in [^18^F]THK-5317 *V*_*T*_s in the striatum and brainstem in 21-month compared to 7-month old animals, whereas significantly higher *V*_*T*_s in the hippocampus, thalamus, and hypothalamus were only found in female animals. Comparable results were recently obtained by Chaney et al. to characterize TgF344-AD (TG) rats [28]. However, contrary to the findings by Chaney et al., immunofluorescent labeling revealed in our study significantly higher total human tau levels in APP/Tau rats compared to ntg animals only at the age of 7 months but not in older age groups. Moreover, tau labeling was slightly higher in the 13-month and markedly reduced in the 21-month old APP/Tau rats. Even if this reduction can most likely be mainly related to the change of the Tau13 antibody batch, it has to be mentioned that Cohen et al. [16] reported a progressive cortical tau pathology from 6- to 16-month of age with an apparent reduction at 26-month of age based on biochemical analysis.

We then attempted to apply the same strategy for the [^11^C]PiB data to the [^18^F]THK-5317 results by excluding tau-negative animals from the analysis. However, this was not feasible for the 21-months age group, as the immunofluorescent labeling results revealed only one tau-positive animal in this group. In the two remaining age groups (7 and 13 months), no correlation was found between IR areas and the respective [^18^F]THK-5317 *V*_*T*_ values. It can be hypothesized that this disagreement between the in vivo PET results and the immunofluorescent labeling results may be related to the previously reported off-target binding of [^18^F]THK-5317 and other quinoline-derived tau radiotracers to monoamine oxidase B (MAO-B) [29, 30]. Therefore, it cannot be excluded that newer generation tau radiotracers [31, 32] would show a better agreement between in vivo and ex vivo results.

In contrast to the study presented by Chaney et al. [28], prodromal neuroinflammation was not found in our APP/Tau rat model as we did not observe any significant differences in GFAP or IBA1 labeling between APP/Tau or ntg rats in all studied age groups. On the other hand, the parent hTau-40/P301L strain of our rat model did not show any evidence of gliosis or inflammation [18], and thus, we speculate that this characteristic is genetically determined.

The motivation behind crossing male McGill-R-Thy1-APP transgenic rats with female hTau-40/P301L rats to generate our new APP/Tau rat model was to enhance brain pathology. According to the “amyloid cascade hypothesis” [33, 34], the deposition of Aβ is the initial pathological trigger in the disease, which subsequently leads to the formation of NFTs, neuronal cell death, and dementia. This hypothesis is supported by data from an APP/Tau transgenic mouse model (TAPP mice) based on double mutant tau (P301L) and mutant APP (APPsw). This model revealed an interaction between APP/Aβ and tau that led to increased NFT formation and distribution in brain regions vulnerable to these lesions [20]. Based on these previous results, we expected that the combined expression of tau and APP/Aβ in the APP/Tau rat model would lead to higher tau and Aβ deposition and aggregation levels than in McGill-R-Thy1-APP transgenic rats. The TAPP mouse model expresses APP from Swedish mutations (Tg2576 mice) and tau from the P301L mutation (JNPL3 mice) and thus from the same mutations as in our rat model, but our rat model additionally expresses APP from the Indiana mutation V717F [15, 20]. Observed differences between studies are therefore unlikely to result from differences in mutations. However, one crucial difference between these two models is the applied protomers to drive transgene expression. While Lewis and colleagues used the prion promoter to drive both APP and tau expression in TAPP mice [20] in our model, APP expression was regulated by the Thy-1 promoter expressing the transgene only in neuronal and specific visceral tissue. In contrast, tau expression was regulated by the prion promoter expressing the transgene ubiquitously [15]. The different promoters cause the expression of the transgenes in different cell types and starting at different time points during development and adulthood. While expression of the prion promoter starts already in post-mitotic cells during embryonal development [35], expression of the Thy-1 promoter starts at early postnatal age [36]. In the here analyzed cross-bred APP/Tau rat model, the expression of tau thus started earlier and more ubiquitously than APP expression, resulting in a different pathological phenotype.

A large body of evidence challenges the amyloid cascade hypothesis as the main event in AD pathogenesis [37]. In our model, the formation of NFTs appeared to precede plaque formation. Schönheit et al. [38] argue that NFTs are formed independently from Aβ and are thus not caused by Aβ in the sense of the amyloid cascade hypothesis. However, as suggested by Fagan et al. [39], we established a weak but positive linear relationship between CSF tau with [^11^C]PiB *BP*_*ND*_ (see Figure 5S D). Animals with higher CSF tau showed a higher [^11^C]PiB accumulation in the cortex. According to Fagan et al., this correlation suggests that increasing amyloid deposition drives neurodegeneration as reflected by CSF tau. This finding would be consistent with results from transgenic mice in which Aβ aggregation/deposition appears to accelerate NFT formation in vivo [40]. On the other hand, in our rat model, tau expression is also regulated by the prion promoter, which activity starts earlier than the Thy-1 promoter of the APP transgene. Thus, in the present model, we observe the early formation of NFTs and, at later time points, enhanced CSF tau levels combined with increased amyloid deposition.

The currently most accepted model in humans describing the spatial-temporal relationship between the Aβ pathway and tau pathophysiology in AD indicates that Aβ may be an upstream pathophysiological event and functions as a trigger of downstream molecular pathways, including tau misfolding, accumulation in tangles, and tau spreading [41]. Therefore, as the order of expression is different from the one observed in humans, this animal model is not suitable for translational research in AD.

### Limitations

Although the study was well planned, it faced some limitations. First, the newly developed double-transgenic APP/Tau rat model exhibited an unstable brain pathohistology. Although all employed APP/Tau rats were positively genotyped twice for tau and APP gene expression, Aβ and tau were not found in all the brain sections by immunofluorescence labeling. This specific characteristic seems to be inherited from the mother hTau-40/P301L genome and limits the use of this animal model for further studies. Second, for some study groups (e.g. ntg 13 months old), we did not reach the planned number of animals due to technical problems with the PET scanner and animals deceased during scanning or surgery. Therefore, statistical analysis between ntg and APP/Tau rats was not possible.

Third, the motivation for this study was that the combined expression of tau and APP/Aβ in this newly developed rat model would lead to higher levels of tau and Aβ deposition and aggregation than in McGill-R-Thy1-APP transgenic rats. However, as we have not included the parent strains (McGill-R-Thy1-APP and hTau-40/P301L rats) in this study analyzed at the same ages, a direct comparison cannot be made.

Finally, the kinetic modeling approaches for analyzing [^11^C]PiB-PET images (SRTM and LRT) were not validated against a blood-based compartmental model, including analysis of radioactive metabolites in plasma and brain.

## Conclusion

We thoroughly characterized a novel APP/Tau rat model using combined PET imaging and immunofluorescence analysis. We observed an age-related increase in [^11^C]PiB and [^18^F]THK-5317 binding in several brain regions in the APP/Tau group but not in the ntg group. Although we revealed a positive correlation between amyloid labeling and [^11^C]PiB regional brain uptake, we observed relatively low human tau and amyloid fibril expression levels and a somewhat unstable brain pathology that questions the utility of this animal model for further studies. Our data highlight the utility of in vivo imaging in evaluating new animal models of AD.

## Supplementary Information

**Additional file 1.**

## Data Availability

The datasets used and/or analyzed during the current study are available from the corresponding author on reasonable request.

## Abbreviations

[^11^C]PiB: ^11^C-labeled Pittsburgh Compound-B
1T2K: One-tissue two-rate constant
2T4K: Two-tissue four-rate constant
AD: Alzheimer’s disease
AIC: Akaike information criterion
APP: Amyloid precursor protein
ARRIVE: Animal Research: Reporting of in Vivo Experiments
Aβ: Amyloid-beta
*BP*_*ND*_: Binding potential
CSF: Cerebrospinal fluid
IF: Input function
IR area: Immunoreactive area
LRT: Logan reference tissue model
MAO-B: Monoamine oxidase B
MR: Magnetic resonance
NFTs: Neurofibrillary tangles
ntg: Non-transgenic
PBS: Phosphate-buffered saline
PET: Positron emission tomography
PFA: Paraformaldehyde
p-tau: Phosphorylated tau
qPCR: Real-time polymerase chain reaction
SD: Standard deviation
SRTM: Simplified reference tissue model
SUV: Standardized uptake value
TACs: Time-activity curves
*V*_*T*_: Volume of distribution
