# Supplementary material for: Characterization of an APP/tau rat model of Alzheimer’s disease by positron emission tomography and immunofluorescent labeling
Source: Alzheimers Res Ther. 2021 Oct 16;13:175. doi: 10.1186/s13195-021-00916-2 (PMC8522096; doi:10.1186/s13195-021-00916-2)

Supplementary Material

Characterization of an APP/Tau rat model of Alzheimer's disease by positron emission tomography and immunofluorescent labeling

Thomas Filip[1,2*], Severin Mairinger[1,3*], Joerg Neddens[4], Michael Sauberer[1,5], Stefanie Flunkert[4], Johann Stanek[1,5], Thomas Wanek[1,5], Nobuyuki Okamura[6], Oliver Langer[1,3,5], Birgit Hutter-Paier[4], Claudia Kuntner[5]

* These authors contributed equally to this work.

[1] *Preclinical Molecular Imaging, AIT Austrian Institute of Technology GmbH, 2444 Seibersdorf, Austria*

[2] *Department of Biomedical Research, Medical University Vienna, Vienna, Austria*

[3] *Department of Clinical Pharmacology, Medical University of Vienna, Vienna, Austria*

[4] *Neuropharmacology, QPS Austria GmbH, Grambach, Austria*

[5] *Department of Biomedical Imaging and Image-guided Therapy, Medical University of Vienna, Vienna, Austria*

[6] *Division of Pharmacology, Faculty of Medicine, Tohoku Medical and Pharmaceutical University, Sendai, Japan*

Results

Evaluation of reference region-based and blood-based kinetic models for [^{18}F]THK-5317 analysis

The [^{18}F]THK-5317 PET time-activity curves (TACs) from the 21-month old animals (females and males) were analyzed by applying reference region-based models with the cerebellum as a reference region (SRTM and LRT) and by applying the one-tissue two-rate constant (1T2K) and the two-tissue four-rate constant (2T4K) blood-based compartment models. Use of the reference region-based models yielded BP_{ND} values equal 0 (SRTM) or <0 (LRT). This is exemplified by PET TACs in the cortex and cerebellum, as shown in Figure 6SA for the SRTM. The cortical TAC (green data points) is below the cerebellar TAC (yellow curve), and thus the calculation of the BP_{ND} is not valid, indicating that the cerebellum is not a suitable reference region in the brain for [^{18}F]THK-5317 analysis.

In the next step, [^{18}F]THK-5317 brain TACs were analyzed using the blood-based models (1T2K and 2T4K models) and Logan graphical analysis. We used a sex-specific metabolite corrected population-based plasma and blood input function (IF) to model brain TACs of APP/Tau and ntg rats. This approach was first validated in a small group of rats (n = 8, 10 brain regions/rat), in which data were additionally modeled using the individual IFs and compared with results of the population-based IF. As shown in Figure 7SA, apart from 1 animal, all other data points showed a good agreement between the V_Ts based on the population-based IF and the V_Ts based on the individual IF (r=0.7316, p<0.0001), which justified the use of the sex-specific metabolite corrected population-based plasma and blood IFs for modeling of [^{18}F]THK-5317 data.

An example of Logan graphical analysis is illustrated in Figure 6SB. The 1T2K model did not fit the data points well (Figure 6SC) by visual inspection, whereas the 2T4K model yielded good fits (Figure 6SD). Moreover, the 2T4K model showed lower Akaike information criterion

(AIC) values for all brain regions than the 1T2K model (data not shown), which supports that the 2T4K model better fitted the data. 2T4K V_Ts showed a slightly better correlation with Logan V_Ts than 1T2K V_Ts (Figure 7SB). Therefore, the 2T4K model and Logan graphical analysis were selected as the preferred kinetic models for [^{18}F]THK-5317 analysis.

Table 1S Number of amyloid- and tau-positive and negative APP/Tau rats based on results from immunofluorescent labeling in the hippocampus of extracted brains. All listed rats were positively genotyped twice for APP and Tau. Animals were considered amyloid- or tau-positive if the hippocampal immunoreactive area (IR area) in the APP/Tau rat brains was > 3x higher than the mean IR area in ntg rat brains.

			Female		Male	
			amyloid			
**Age**			pos	neg	pos	neg
**7 months**	tau	pos	-	3	1	1
		neg	-	2	1	2
**13 months**		pos	4	-	6	2
		neg	2	2	3	1
**21 months**		pos	-	-	1	-
		neg	6	-	6	1

Table 2S

Binding potential (BP_{ND}) of [^{11}C]PiB in brain regions of female and male APP/Tau rats obtained with the Simplified Reference Tissue Model (SRTM) and Logan reference tissue (LRT) model with the cerebellum as reference region. Outcome parameters (BP_{ND} (SRTM), BP_{ND} (LRT), and AIC (Akaike information criterion)) are given as mean ± SD averaged over all animals per age group. The value in parentheses represents the precision of parameter estimates (expressed as mean coefficient of variation given in percent).

**PIB**	**7 months (n=6)**			**13 months (n=11)**			**21 months (n=12)**		
	***BP*$_{ND}$ (SRTM)**	**AIC**	***BP*$_{ND}$ (LRT)**	***BP*$_{ND}$ (SRTM)**	**AIC**	***BP*$_{ND}$ (LRT)**	***BP*$_{ND}$ (SRTM)**	**AIC**	***BP*$_{ND}$ (LRT)**
Frontal Cortex	0.22 ± 0.06 (27%)	30.91 ± 19.02	0.29 ± 0.05 (17%)	0.28 ± 0.08 (29%)	38.64 ± 16.13	0.34 ± 0.06 (18%)	0.33 ± 0.14 (42%)	21.75 ± 20.90	0.39 ± 0.12 (32%)
Somatosensory	0.22 ± 0.06 (25%)	35.98 ± 18.46	0.29 ± 0.06 (22%)	0.30 ± 0.09 (31%)	37.22 ± 12.82	0.36 ± 0.06 (17%)	0.34 ± 0.11 (31%)	22.64 ± 23.05	0.42 ± 0.15 (36%)
Cortex	0.32 ± 0.06 (19%)	19.04 ± 21.58	0.38 ± 0.08 (21%)	0.37 ± 0.07 (19%)	20.66 ± 12.72	0.42 ± 0.05 (12%)	0.40 ± 0.08 (19%)	19.02 ± 17.37	0.47 ± 0.16 (35%)
Cortex all	0.27 ± 0.06 (22%)	20.38 ± 17.52	0.33 ± 0.07 (20%)	0.33 ± 0.07 (22%)	18.66 ± 11.00	0.39 ± 0.05 (14%)	0.37 ± 0.09 (25%)	10.90 ± 19.66	0.44 ± 0.16 (36%)
Hippocampus	0.71 ± 0.05 (7%)	32.95 ± 29.01	0.79 ± 0.13 (16%)	0.75 ± 0.12 (16%)	43.71 ± 11.11	0.79 ± 0.10 (13%)	0.79 ± 0.17 (22%)	31.44 ± 27.26	0.86 ± 0.23 (27%)
Thalamus	0.94 ± 0.09 (10%)	40.94 ± 16.22	1.00 ± 0.10 (10%)	0.92 ± 0.13 (14%)	48.59 ± 12.02	1.01 ± 0.11 (11%)	0.97 ± 0.14 (14%)	35.72 ± 14.68	1.08 ± 0.14 (13%)
Striatum	0.43 ± 0.10 (23%)	36.94 ± 18.10	0.50 ± 0.08 (17%)	0.41 ± 0.07 (17%)	35.92 ± 17.92	0.50 ± 0.06 (12%)	0.53 ± 0.10 (19%)	27.64 ± 18.27	0.62 ± 0.12 (19%)
Hypothalamus	1.04 ± 0.14 (13%)	58.01 ± 19.88	1.09 ± 0.28 (25%)	1.10 ± 0.10 (9%)	57.07 ± 11.55	1.10 ± 0.10 (9%)	1.14 ± 0.23 (20%)	48.42 ± 24.23	1.12 ± 0.23 (20%)
Brainstem	0.38 ± 0.06 (15%)	33.33 ± 27.41	0.41 ± 0.10 (23%)	0.39 ± 0.05 (13%)	34.73 ± 18.59	0.39 ± 0.05 (14%)	0.41 ± 0.10 (24%)	23.85 ± 23.46	0.43 ± 0.13 (31%)

Table 3S

Biodistribution analysis of [^{18}F]THK-5317 in the brain, CSF, blood, and plasma of female and male APP/Tau and ntg rats measured at 60 min after radiotracer injection. Brain values were determined after transcardial perfusion. Data are presented as standardized uptake values (SUV; mean ± SD), including the number of analyzed animals (n).

	**7 month**		**13 month**		**21 month**	
**male**	**ntg**	**APP/Tau**	**ntg**	**APP/Tau**	**ntg**	**APP/Tau**
**Brain**	0.35 ± 0.05 (5)	0.35 ± 0.04 (4)	n.a.	0.33 ± 0.14 (10)	0.23 ± 0.04 (2)	0.40 ± 0.06 (8)
**CSF**	n.a.	n.a.	n.a.	0.02 ± 0.01 (10)	0.03 ± 0.02 (2)	0.02 ± 0.01 (8)
**Blood**	0.65 ± 0.12 (5)	0.74 ± 0.04 (4)	n.a.	0.63 ± 0.29 (10)	0.23 ± 0.04 (2)	0.50 ± 0.09 (8)
**Plasma**	1.08 ± 0.21 (5)	1.01 ± 0.19 (4)	n.a.	0.69 ± 0.15 (10)	0.37 ± 0.14 (2)	0.65 ± 0.20 (8)
**female**	**ntg**	**APP/Tau**	**ntg**	**APP/Tau**	**ntg**	**APP/Tau**
**Brain**	0.34 ± 0.06 (4)	0.28 ± 0.02 (4)	0.31 ± 0.01 (2)	0.29 ± 0.11 (7)	0.36 ± 0.06 (2)	0.36 ± 0.09 (6)
**CSF**	n.a.	n.a.	0.03 ± 0.02 (2)	0.01 ± 0.01 (7)	0.02 ± 0.01 (2)	0.03 ± 0.03 (6)
**Blood**	0.23 ± 0.08 (4)	0.28 ± 0.08 (5)	0.70 ± 0.31 (2)	0.38 ± 0.23 (7)	0.33 ± 0.07 (2)	0.49 ± 0.27 (6)
**Plasma**	0.26 ± 0.09 (4)	0.24 ± 0.02 (5)	0.48 ± 0.15 (2)	0.32 ± 0.15 (7)	0.39 ± 0.14 (2)	0.66 ± 0.54 (6)

Table 4S

Outcome parameters of [^{18}F]THK-5317 kinetic modeling in brain regions of female APP/Tau and ntg rats using the two-tissue four-rate constant (2T4K) model and Logan graphical analysis. Outcome parameters (K_1, k_2, k_3, k_4, V_T, K_1/k_2, k_3/k_4, and AIC (Akaike information criterion)) are given as mean ± SD averaged over all animals per age group. The value in parentheses represents the precision of parameter estimates (expressed as mean coefficient of variation given in percent).

	K_1 [ml/cc/min]	k_2 [1/min]	k_3 [1/min]	k_4 [1/min]	V_T [ml/cc]	K_1/k_2 [ml/ccm]	k_3/k_4	AIC	V_T (Logan) [ml/cc]	AIC
**7 months female APP/Tau (n=7)**										
Frontal cortex	1.71 ± 0.39 (23%)	0.78 ± 0.19 (24%)	0.28 ± 0.12 (43%)	0.17 ± 0.04 (24%)	5.76 ± 0.65 (11%)	2.26 ± 0.41 (18%)	1.61 ± 0.43 (27%)	35.59 ± 12.08	5.98 ± 0.64 (11%)	54.37 ± 3.90
Som.sen. cortex	1.96 ± 0.69 (35%)	0.90 ± 0.67 (75%)	0.29 ± 0.22 (77%)	0.15 ± 0.04 (24%)	6.59 ± 0.64 (10%)	2.54 ± 0.71 (28%)	1.80 ± 1.00 (55%)	30.34 ± 11.12	6.83 ± 0.66 (10%)	54.24 ± 4.77
Cortex	1.65 ± 0.47 (28%)	0.84 ± 0.51 (61%)	0.29 ± 0.21 (70%)	0.15 ± 0.04 (25%)	5.86 ± 0.64 (11%)	2.22 ± 0.60 (27%)	1.83 ± 0.92 (50%)	22.74 ± 13.05	6.09 ± 0.63 (10%)	52.95 ± 3.00
Cortex all	1.68 ± 0.40 (24%)	0.75 ± 0.30 (40%)	0.26 ± 0.14 (54%)	0.15 ± 0.03 (23%)	6.02 ± 0.63 (11%)	2.37 ± 0.51 (21%)	1.64 ± 0.60 (36%)	23.65 ± 11.17	6.24 ± 0.63 (10%)	52.33 ± 3.26
Hippocampus	2.77 ± 1.95 (71%)	1.89 ± 2.72 (144%)	0.45 ± 0.40 (90%)	0.15 ± 0.04 (26%)	7.23 ± 0.81 (11%)	2.45 ± 1.12 (46%)	2.67 ± 2.17 (81%)	40.71 ± 10.13	7.58 ± 0.70 (9%)	49.92 ± 2.55
Thalamus	2.47 ± 1.00 (40%)	0.94 ± 0.75 (80%)	0.30 ± 0.20 (67%)	0.16 ± 0.05 (30%)	7.97 ± 0.95 (12%)	3.23 ± 1.14 (35%)	1.74 ± 1.06 (61%)	43.12 ± 11.71	8.12 ± 0.94 (12%)	52.23 ± 3.81
Striatum	2.05 ± 0.60 (30%)	0.96 ± 0.58 (61%)	0.39 ± 0.30 (76%)	0.17 ± 0.05 (30%)	7.12 ± 0.94 (13%)	2.74 ± 1.24 (45%)	2.07 ± 1.29 (62%)	37.04 ± 11.19	7.33 ± 0.94 (13%)	52.94 ± 4.89
Hypothalamus	3.22 ± 2.00 (62%)	2.48 ± 2.88 (116%)	0.47 ± 0.40 (85%)	0.16 ± 0.03 (22%)	6.49 ± 0.85 (13%)	2.16 ± 1.10 (51%)	2.74 ± 1.92 (70%)	49.28 ± 10.94	6.88 ± 0.82 (12%)	59.06 ± 5.00
Cerebellum	2.52 ± 0.67 (26%)	0.95 ± 0.35 (37%)	0.20 ± 0.09 (43%)	0.13 ± 0.03 (21%)	6.68 ± 0.75 (11%)	2.76 ± 0.52 (19%)	1.46 ± 0.37 (25%)	31.33 ± 10.76	6.96 ± 0.75 (11%)	53.00 ± 2.71
Brainstem	2.51 ± 1.06 (42%)	1.18 ± 0.85 (72%)	0.37 ± 0.17 (45%)	0.15 ± 0.02 (10%)	7.91 ± 1.06 (13%)	2.42 ± 0.65 (27%)	2.46 ± 1.02 (42%)	28.00 ± 13.93	8.24 ± 1.04 (13%)	52.12 ± 4.37
**13 months female APP/Tau (n=7)**										
	**K_1 [ml/cc/min]**	**k_2 [1/min]**	**k_3 [1/min]**	**k_4 [1/min]**	**V_T [ml/cc]**	**K_1/k_2 [ml/ccm]**	**k_3/k_4**	**AIC**	**V_T (Logan) [ml/cc]**	**AIC**
Frontal cortex	1.58 ± 0.25 (16%)	0.64 ± 0.28 (43%)	0.28 ± 0.33 (120%)	0.15 ± 0.08 (54%)	6.12 ± 0.66 (11%)	2.78 ± 0.98 (35%)	1.46 ± 0.89 (61%)	38.18 ± 4.45	6.36 ± 0.50 (8%)	53.39 ± 3.60
Som.sen. cortex	1.63 ± 0.41 (25%)	0.57 ± 0.39 (67%)	0.17 ± 0.16 (96%)	0.12 ± 0.03 (23%)	7.00 ± 0.68 (10%)	3.33 ± 0.88 (26%)	1.29 ± 0.84 (65%)	38.63 ± 8.78	7.21 ± 0.64 (9%)	52.81 ± 2.43

Cortex	1.51 ± 0.17 (11%)	0.60 ± 0.12 (20%)	0.22 ± 0.21 (93%)	0.13 ± 0.06 (45%)	6.36 ± 0.48 (8%)	2.62 ± 0.60 (23%)	1.54 ± 0.58 (38%)	25.15 ± 8.69	6.57 ± 0.40 (6%)	52.17 ± 5.19
Cortex all	1.52 ± 0.25 (16%)	0.53 ± 0.12 (23%)	0.17 ± 0.09 (54%)	0.13 ± 0.05 (42%)	6.47 ± 0.54 (8%)	2.91 ± 0.53 (18%)	1.26 ± 0.31 (25%)	20.92 ± 10.91	6.66 ± 0.47 (7%)	53.70 ± 1.85
Hippocampus	1.78 ± 0.34 (19%)	0.64 ± 0.25 (39%)	0.29 ± 0.29 (103%)	0.13 ± 0.06 (50%)	8.08 ± 0.88 (11%)	3.11 ± 1.11 (36%)	1.87 ± 0.97 (52%)	41.68 ± 13.54	8.32 ± 0.71 (8%)	54.45 ± 1.79
Thalamus	2.05 ± 0.52 (25%)	0.52 ± 0.21 (40%)	0.14 ± 0.07 (52%)	0.12 ± 0.02 (16%)	8.74 ± 0.65 (7%)	4.32 ± 1.04 (24%)	1.12 ± 0.49 (43%)	47.42 ± 15.30	8.93 ± 0.68 (8%)	56.47 ± 3.80
Striatum	1.73 ± 0.38 (22%)	0.54 ± 0.28 (52%)	0.19 ± 0.15 (78%)	0.14 ± 0.04 (27%)	7.81 ± 0.60 (8%)	3.64 ± 0.94 (26%)	1.29 ± 0.67 (52%)	39.11 ± 9.10	7.99 ± 0.54 (7%)	54.19 ± 2.22
Hypothalamus	3.03 ± 2.38 (79%)	2.15 ± 2.80 (130%)	0.45 ± 0.41 (90%)	0.14 ± 0.05 (38%)	7.10 ± 0.29 (4%)	2.43 ± 1.20 (49%)	2.73 ± 2.04 (75%)	53.14 ± 13.97	7.43 ± 0.28 (4%)	54.54 ± 7.03
Cerebellum	2.17 ± 0.44 (20%)	0.72 ± 0.19 (26%)	0.14 ± 0.05 (36%)	0.11 ± 0.03 (30%)	7.15 ± 0.59 (8%)	3.07 ± 0.38 (13%)	1.34 ± 0.20 (15%)	24.82 ± 6.04	7.36 ± 0.55 (7%)	50.97 ± 2.57
Brainstem	2.15 ± 0.70 (33%)	1.06 ± 0.85 (80%)	0.45 ± 0.41 (91%)	0.14 ± 0.05 (39%)	8.51 ± 0.69 (8%)	2.57 ± 0.96 (38%)	2.84 ± 1.67 (59%)	23.79 ± 14.01	8.91 ± 0.57 (6%)	51.31 ± 4.92
**21 months female APP/Tau (n=6)**										
	**K_1 [ml/cc/min]**	**k_2 [1/min]**	**k_3 [1/min]**	**k_4 [1/min]**	**V_T [ml/cc]**	**K_1/k_2 [ml/ccm]**	**k_3/k_4**	**AIC**	**V_T (Logan) [ml/cc]**	**AIC**
Frontal cortex	1.79 ± 0.56 (31%)	0.60 ± 0.3 (50%)	0.19 ± 0.15 (82%)	0.13 ± 0.06 (48%)	7.06 ± 0.99 (14%)	3.35 ± 0.96 (29%)	1.23 ± 0.55 (45%)	30.76 ± 7.19	7.31 ± 0.91 (12%)	54.52 ± 2.82
Som.sen. cortex	1.80 ± 0.58 (32%)	0.55 ± 0.32 (58%)	0.16 ± 0.13 (81%)	0.11 ± 0.05 (45%)	8.31 ± 1.03 (12%)	3.85 ± 1.25 (32%)	1.32 ± 0.65 (49%)	25.58 ± 11.61	8.55 ± 1.05 (12%)	54.44 ± 3.97
Cortex	1.60 ± 0.46 (29%)	0.54 ± 0.31 (58%)	0.15 ± 0.13 (86%)	0.11 ± 0.05 (47%)	7.32 ± 1.00 (14%)	3.48 ± 1.15 (33%)	1.26 ± 0.61 (48%)	19.80 ± 10.14	7.53 ± 0.94 (12%)	53.09 ± 2.95
Cortex all	1.68 ± 0.51 (31%)	0.54 ± 0.29 (54%)	0.15 ± 0.12 (79%)	0.11 ± 0.05 (44%)	7.50 ± 0.96 (13%)	3.57 ± 1.05 (30%)	1.23 ± 0.55 (45%)	15.64 ± 5.89	7.72 ± 0.94 (12%)	52.56 ± 2.89
Hippocampus	1.90 ± 0.53 (28%)	0.53 ± 0.23 (44%)	0.17 ± 0.07 (41%)	0.12 ± 0.03 (28%)	9.24 ± 1.16 (13%)	3.84 ± 0.81 (21%)	1.47 ± 0.48 (32%)	38.73 ± 13.55	9.57 ± 1.25 (13%)	51.01 ± 2.75
Thalamus	2.28 ± 0.67 (29%)	0.58 ± 0.28 (49%)	0.19 ± 0.13 (67%)	0.12 ± 0.05 (41%)	10.10 ± 1.29 (13%)	4.61 ± 1.65 (36%)	1.38 ± 0.66 (48%)	41.49 ± 12.38	10.32 ± 1.35 (13%)	55.71 ± 5.01
Striatum	2.15 ± 0.77 (36%)	0.88 ± 1.08 (123%)	0.31 ± 0.41 (133%)	0.13 ± 0.06 (46%)	9.42 ± 1.44 (15%)	4.12 ± 1.87 (45%)	1.90 ± 1.74 (92%)	32.08 ± 10.37	9.69 ± 1.17 (12%)	52.65 ± 4.97
Hypothalamus	2.27 ± 0.81 (36%)	0.83 ± 0.56 (68%)	0.31 ± 0.22 (70%)	0.15 ± 0.06 (42%)	8.82 ± 1.61 (18%)	3.34 ± 1.63 (49%)	1.92 ± 0.85 (44%)	55.75 ± 10.50	9.07 ± 1.40 (15%)	56.22 ± 2.59
Cerebellum	2.34 ± 0.69 (29%)	0.73 ± 0.37 (50%)	0.15 ± 0.09 (62%)	0.10 ± 0.04 (37%)	7.94 ± 1.08 (14%)	3.47 ± 0.80 (23%)	1.35 ± 0.40 (30%)	26.22 ± 7.48	8.18 ± 0.98 (12%)	53.36 ± 3.79
Brainstem	2.17 ± 0.69 (32%)	0.80 ± 0.52 (65%)	0.31 ± 0.21 (70%)	0.13 ± 0.04 (31%)	9.73 ± 1.57 (16%)	3.41 ± 1.38 (40%)	2.20 ± 1.09 (50%)	30.81 ± 5.41	10.10 ± 1.45 (14%)	52.22 ± 5.14
**female ntg (n=15)**										
	**K_1 [ml/cc/min]**	**k_2 [1/min]**	**k_3 [1/min]**	**k_4 [1/min]**	**V_T [ml/cc]**	**K_1/k_2 [ml/ccm]**	**k_3/k_4**	**AIC**	**V_T (Logan) [ml/cc]**	**AIC**
Frontal cortex	1.70 ± 0.53 (31%)	0.72 ± 0.59 (82%)	0.24 ± 0.22 (91%)	0.15 ± 0.05 (35%)	6.14 ± 0.66 (11%)	2.89 ± 0.92 (32%)	1.34 ± 0.89 (66%)	34.82 ± 16.29	6.33 ± 0.65 (10%)	53.10 ± 4.27

Som.sen. cortex	1.75 ± 0.52 (29%)	0.66 ± 0.50 (77%)	0.22 ± 0.21 (100%)	0.14 ± 0.04 (32%)	6.92 ± 0.89 (13%)	3.22 ± 0.93 (29%)	1.35 ± 0.84 (62%)	36.58 ± 15.23	7.10 ± 0.85 (12%)	52.93 ± 5.17
Cortex	1.57 ± 0.46 (29%)	0.68 ± 0.42 (61%)	0.22 ± 0.17 (77%)	0.14 ± 0.04 (32%)	6.13 ± 0.65 (11%)	2.75 ± 0.84 (31%)	1.45 ± 0.81 (56%)	31.20 ± 17.22	6.34 ± 0.66 (10%)	53.40 ± 3.01
Cortex all	1.61 ± 0.43 (27%)	0.63 ± 0.35 (56%)	0.21 ± 0.15 (73%)	0.14 ± 0.04 (31%)	6.32 ± 0.69 (11%)	2.91 ± 0.81 (28%)	1.33 ± 0.67 (50%)	28.38 ± 15.89	6.52 ± 0.69 (11%)	52.77 ± 3.44
Hippocampus	2.17 ± 1.19 (55%)	1.22 ± 1.70 (140%)	0.34 ± 0.39 (116%)	0.14 ± 0.05 (33%)	7.61 ± 0.90 (12%)	3.20 ± 1.39 (43%)	2.11 ± 2.10 (100%)	41.81 ± 13.05	7.93 ± 0.86 (11%)	51.76 ± 4.83
Thalamus	2.37 ± 1.07 (45%)	0.87 ± 0.69 (80%)	0.32 ± 0.29 (90%)	0.16 ± 0.04 (27%)	8.29 ± 0.92 (11%)	3.56 ± 1.36 (38%)	1.76 ± 1.39 (79%)	41.04 ± 11.51	8.53 ± 0.99 (12%)	58.40 ± 19.72
Striatum	2.19 ± 1.23 (56%)	1.17 ± 1.90 (162%)	0.38 ± 0.37 (99%)	0.17 ± 0.05 (28%)	7.61 ± 0.87 (11%)	3.06 ± 1.29 (42%)	2.13 ± 2.11 (99%)	36.52 ± 15.22	7.84 ± 0.91 (12%)	51.90 ± 3.65
Hypothalamus	2.78 ± 1.72 (62%)	1.88 ± 2.51 (133%)	0.40 ± 0.51 (127%)	0.14 ± 0.04 (31%)	6.86 ± 0.83 (12%)	2.78 ± 1.34 (48%)	2.36 ± 2.33 (99%)	51.04 ± 15.23	7.18 ± 0.93 (13%)	54.95 ± 6.35
Cerebellum	2.39 ± 0.79 (33%)	0.91 ± 0.62 (68%)	0.21 ± 0.16 (75%)	0.13 ± 0.05 (36%)	6.86 ± 0.78 (11%)	3.02 ± 0.84 (28%)	1.40 ± 0.55 (39%)	37.15 ± 14.60	7.19 ± 0.70 (10%)	52.07 ± 3.53
Brainstem	2.25 ± 0.71 (32%)	0.92 ± 0.50 (55%)	0.37 ± 0.23 (62%)	0.15 ± 0.03 (20%)	8.35 ± 1.00 (12%)	2.90 ± 1.16 (40%)	2.22 ± 1.03 (46%)	35.95 ± 11.86	8.69 ± 0.97 (11%)	52.22 ± 3.74

Table 5S

Outcome parameters of [^{18}F]THK-5317 kinetic modeling in brain regions of male APP/Tau and ntg rats using the two-tissue four-rate constant (2T4K) model and Logan graphical analysis. Outcome parameters (K_1, k_2, k_3, k_4, V_T, K_1/k_2, k_3/k_4, and AIC (Akaike information criterion)) are given as mean ± SD averaged over all animals per age group. The value in parentheses represents the precision of parameter estimates (expressed as mean coefficient of variation given in percent).

	**7 months male APP/Tau (n=7)**									
	***K_1* [ml/cc/min]**	***k_2* [1/min]**	***k_3* [1/min]**	***k_4* [1/min]**	***V_T* [ml/cc]**	***K_1/k_2* [ml/ccm]**	***k_3/k_4***	**AIC**	***V_T* (Logan) [ml/cc]**	**AIC**
Frontal cortex	2.55 ± 1.17 (46%)	1.05 ± 1.65 (158%)	0.39 ± 0.72 (186%)	0.18 ± 0.16 (86%)	7.97 ± 0.99 (12%)	4.91 ± 2.17 (44%)	1.21 ± 1.75 (145%)	28.89 ± 13.44	8.21 ± 0.8 (10%)	52.47 ± 4.59
Som.sen. cortex	2.95 ± 1.63 (55%)	1.56 ± 2.83 (181%)	0.65 ± 0.99 (152%)	0.28 ± 0.31 (110%)	8.72 ± 1.11 (13%)	4.41 ± 1.92 (44%)	1.79 ± 2.45 (137%)	43.96 ± 8.48	9.19 ± 0.96 (10%)	54.49 ± 2.30
Cortex	2.72 ± 1.83 (67%)	1.53 ± 2.86 (187%)	0.37 ± 0.73 (199%)	0.14 ± 0.10 (70%)	7.72 ± 1.00 (13%)	4.37 ± 1.92 (44%)	1.45 ± 2.04 (141%)	27.45 ± 8.59	7.97 ± 0.78 (10%)	55.13 ± 3.55
Cortex all	2.72 ± 1.68 (62%)	1.43 ± 2.69 (188%)	0.39 ± 0.82 (212%)	0.14 ± 0.11 (76%)	8.09 ± 1.04 (13%)	4.78 ± 2.06 (43%)	1.42 ± 2.18 (154%)	22.26 ± 15.32	8.32 ± 0.82 (10%)	53.41 ± 4.38
Hippocampus	3.33 ± 0.83 (25%)	2.30 ± 2.77 (121%)	1.45 ± 2.26 (156%)	0.23 ± 0.13 (57%)	8.92 ± 0.97 (11%)	3.41 ± 2.34 (68%)	4.14 ± 5.44 (131%)	45.01 ± 11.01	9.55 ± 0.96 (10%)	53.96 ± 2.32
Thalamus	4.75 ± 2.23 (47%)	3.43 ± 3.36 (98%)	1.86 ± 2.11 (113%)	0.37 ± 0.25 (66%)	9.92 ± 0.78 (8%)	3.43 ± 2.85 (83%)	4.96 ± 5.42 (109%)	50.06 ± 6.66	10.61 ± 0.94 (9%)	55.03 ± 6.58
Striatum	2.69 ± 0.99 (37%)	0.87 ± 1.09 (125%)	0.39 ± 0.53 (138%)	0.20 ± 0.12 (62%)	9.60 ± 1.09 (11%)	5.33 ± 2.79 (52%)	1.41 ± 1.68 (119%)	36.65 ± 12.95	9.91 ± 0.82 (8%)	55.27 ± 1.72
Hypothalamus	5.46 ± 1.69 (31%)	4.69 ± 3.09 (66%)	1.72 ± 1.54 (90%)	0.30 ± 0.07 (23%)	8.00 ± 0.68 (8%)	1.84 ± 1.39 (76%)	5.23 ± 3.59 (69%)	56.30 ± 14.16	9.01 ± 0.78 (9%)	57.72 ± 6.40
Cerebellum	3.82 ± 1.90 (50%)	1.07 ± 1.22 (114%)	0.20 ± 0.28 (141%)	0.16 ± 0.11 (69%)	8.72 ± 1.11 (13%)	4.74 ± 1.26 (27%)	0.96 ± 0.53 (55%)	35.07 ± 10.79	9.22 ± 0.97 (10%)	54.27 ± 3.20
Brainstem	2.84 ± 0.86 (30%)	0.62 ± 0.54 (87%)	0.18 ± 0.25 (138%)	0.14 ± 0.08 (58%)	10.59 ± 1.14 (11%)	5.85 ± 1.93 (33%)	0.99 ± 0.66 (67%)	27.30 ± 10.80	10.93 ± 0.91 (8%)	53.88 ± 3.31
	**13 months male APP/Tau (n=10)**									
	***K_1* [ml/cc/min]**	***k_2* [1/min]**	***k_3* [1/min]**	***k_4* [1/min]**	***V_T* [ml/cc]**	***K_1/k_2* [ml/ccm]**	***k_3/k_4***	**AIC**	***V_T* (Logan) [ml/cc]**	**AIC**
Frontal cortex	2.35 ± 0.44 (19%)	0.65 ± 0.42 (64%)	0.35 ± 0.44 (125%)	0.18 ± 0.10 (53%)	9.02 ± 0.64 (7%)	4.76 ± 2.18 (46%)	1.41 ± 1.37 (97%)	32.82 ± 15.26	9.44 ± 0.55 (6%)	53.52 ± 3.21
Som.sen. cortex	2.07 ± 0.35 (17%)	0.32 ± 0.06 (20%)	0.06 ± 0.03 (54%)	0.09 ± 0.02 (27%)	10.29 ± 0.73 (7%)	6.57 ± 0.96 (15%)	0.59 ± 0.16 (27%)	37.95 ± 13.15	10.35 ± 0.66 (6%)	54.08 ± 3.63

Cortex	1.97 ± 0.36 (18%)	0.38 ± 0.14 (37%)	0.08 ± 0.08 (94%)	0.10 ± 0.04 (39%)	8.92 ± 0.53 (6%)	5.44 ± 1.06 (19%)	0.69 ± 0.34 (49%)	28.34 ± 14.84	9.09 ± 0.39 (4%)	54.54 ± 3.85
Cortex all	2.03 ± 0.37 (18%)	0.36 ± 0.11 (31%)	0.08 ± 0.07 (87%)	0.10 ± 0.04 (39%)	9.30 ± 0.56 (6%)	5.81 ± 1.07 (18%)	0.64 ± 0.28 (44%)	17.58 ± 20.04	9.46 ± 0.49 (5%)	55.53 ± 2.05
Hippocampus	2.99 ± 1.83 (61%)	1.14 ± 2.14 (187%)	0.24 ± 0.42 (173%)	0.13 ± 0.03 (27%)	10.43 ± 0.78 (7%)	5.58 ± 2.01 (36%)	1.51 ± 2.18 (144%)	52.24 ± 10.96	10.79 ± 0.63 (6%)	55.87 ± 3.06
Thalamus	3.22 ± 1.74 (54%)	1.33 ± 2.7. (203%)	0.46 ± 1.00 (216%)	0.15 ± 0.08 (53%)	11.57 ± 0.92 (8%)	6.92 ± 3.12 (45%)	1.86 ± 3.54 (190%)	44.58 ± 15.25	11.88 ± 0.77 (6%)	55.02 ± 2.23
Striatum	2.18 ± 0.43 (20%)	0.31 ± 0.11 (35%)	0.07 ± 0.08 (107%)	0.10 ± 0.04 (45%)	11.47 ± 0.85 (7%)	7.38 ± 1.57 (21%)	0.62 ± 0.38 (61%)	41.58 ± 14.25	11.50 ± 0.84 (7%)	55.35 ± 2.79
Hypothalamus	3.27 ± 2.02 (62%)	1.03 ± 1.44 (140%)	0.27 ± 0.36 (135%)	0.15 ± 0.08 (52%)	10.07 ± 0.46 (5%)	5.24 ± 2.18 (41%)	1.39 ± 1.45 (104%)	57.95 ± 10.90	10.44 ± 0.62 (6%)	57.45 ± 5.32
Cerebellum	3.06 ± 0.69 (22%)	0.57 ± 0.22 (39%)	0.09 ± 0.07 (81%)	0.10 ± 0.04 (46%)	9.92 ± 0.68 (7%)	5.67 ± 1.07 (19%)	0.79 ± 0.26 (33%)	24.49 ± 19.55	10.20 ± 0.57 (6%)	54.70 ± 1.48
Brainstem	3.00 ± 0.62 (21%)	0.67 ± 0.40 (60%)	0.26 ± 0.19 (73%)	0.17 ± 0.05 (29%)	11.80 ± 0.87 (7%)	5.39 ± 1.96 (36%)	1.44 ± 0.82 (57%)	28.71 ± 9.56	12.33 ± 0.58 (5%)	53.97 ± 4.18
**21 months male APP/Tau (n=8)**										
	**K_1 [ml/cc/min]**	**k_2 [1/min]**	**k_3 [1/min]**	**k_4 [1/min]**	**V_T [ml/cc]**	**K_1/k_2 [ml/ccm]**	**k_3/k_4**	**AIC**	**V_T (Logan) [ml/cc]**	**AIC**
Frontal cortex	2.44 ± 1.03 (42%)	0.91 ± 1.34 (147%)	0.41 ± 0.71 (174%)	0.14 ± 0.11 (77%)	10.38 ± 2.34 (23%)	6.72 ± 3.52 (52%)	1.44 ± 2.1 (146%)	29.73 ± 7.91	10.60 ± 2.14 (20%)	52.51 ± 4.58
Som.sen. cortex	2.11 ± 0.63 (30%)	0.36 ± 0.36 (101%)	0.09 ± 0.15 (159%)	0.11 ± 0.06 (56%)	11.69 ± 2.66 (23%)	7.81 ± 2.56 (33%)	0.63 ± 0.54 (86%)	22.24 ± 16.37	11.75 ± 2.51 (21%)	53.97 ± 3.71
Cortex	2.06 ± 0.64 (31%)	0.48 ± 0.58 (121%)	0.14 ± 0.24 (171%)	0.12 ± 0.06 (53%)	10.17 ± 2.14 (21%)	6.42 ± 2.22 (35%)	0.82 ± 0.88 (107%)	26.00 ± 18.78	10.34 ± 2.00 (19%)	53.00 ± 3.49
Cortex all	2.14 ± 0.73 (34%)	0.51 ± 0.72 (141%)	0.15 ± 0.29 (195%)	0.12 ± 0.07 (61%)	10.59 ± 2.30 (22%)	6.96 ± 2.45 (35%)	0.79 ± 0.98 (124%)	16.37 ± 15.19	10.74 ± 2.14 (20%)	52.46 ± 3.71
Hippocampus	2.88 ± 1.77 (61%)	1.44 ± 2.69 (186%)	0.50 ± 0.80 (162%)	0.12 ± 0.08 (68%)	12.60 ± 2.93 (23%)	6.73 ± 4.35 (65%)	2.70 ± 4.36 (161%)	37.97 ± 12.26	12.81 ± 2.55 (20%)	54.15 ± 6.26
Thalamus	4.17 ± 2.69 (65%)	2.85 ± 3.69 (130%)	0.82 ± 1.11 (136%)	0.14 ± 0.06 (44%)	13.29 ± 2.86 (22%)	7.45 ± 5.60 (75%)	4.15 ± 5.56 (134%)	43.24 ± 8.69	13.60 ± 2.62 (19%)	55.14 ± 5.85
Striatum	2.74 ± 1.77 (64%)	1.26 ± 2.73 (216%)	0.39 ± 0.85 (216%)	0.13 ± 0.07 (54%)	13.03 ± 2.68 (21%)	8.00 ± 4.29 (54%)	2.05 ± 4.08 (199%)	31.91 ± 12.44	13.18 ± 2.36 (18%)	52.60 ± 4.98
Hypothalamus	5.58 ± 2.22 (40%)	4.26 ± 3.42 (80%)	1.27 ± 1.04 (82%)	0.21 ± 0.04 (19%)	11.35 ± 2.18 (19%)	2.94 ± 2.67 (91%)	6.28 ± 5.42 (86%)	56.30 ± 6.69	11.95 ± 2.22 (19%)	55.23 ± 8.03
Cerebellum	3.64 ± 1.85 (51%)	1.11 ± 1.69 (152%)	0.27 ± 0.48 (179%)	0.14 ± 0.07 (54%)	11.20 ± 2.64 (24%)	6.12 ± 2.83 (46%)	1.31 ± 1.58 (121%)	34.71 ± 14.06	11.72 ± 2.28 (19%)	53.38 ± 3.52
Brainstem	3.19 ± 0.97 (30%)	0.81 ± 0.95 (118%)	0.35 ± 0.40 (113%)	0.17 ± 0.07 (38%)	13.76 ± 2.94 (21%)	6.75 ± 3.31 (49%)	1.62 ± 1.43 (88%)	28.54 ± 13.94	14.23 ± 2.66 (19%)	53.08 ± 3.42
**male ntg (n=12)**										
	**K_1 [ml/cc/min]**	**k_2 [1/min]**	**k_3 [1/min]**	**k_4 [1/min]**	**V_T [ml/cc]**	**K_1/k_2 [ml/ccm]**	**k_3/k_4**	**AIC**	**V_T (Logan) [ml/cc]**	**AIC**
Frontal cortex	2.89 ± 1.57 (54%)	1.11 ± 2.04 (184%)	0.30 ± 0.51 (168%)	0.17 ± 0.09 (52%)	8.93 ± 1.42 (16%)	5.14 ± 2.18 (42%)	1.27 ± 1.71 (135%)	34.36 ± 13.41	9.25 ± 1.28 (14%)	53.42 ± 3.70

Som.sen. cortex	2.69 ± 1.41 (52%)	1.11 ± 2.19 (196%)	0.36 ± 0.76 (209%)	0.14 ± 0.08 (60%)	9.63 ± 1.67 (17%)	5.16 ± 2.17 (42%)	1.57 ± 2.34 (149%)	39.46 ± 13.63	9.94 ± 1.62 (16%)	54.75 ± 4.18
Cortex	2.36 ± 1.14 (49%)	1.07 ± 2.11 (198%)	0.25 ± 0.49 (197%)	0.13 ± 0.06 (50%)	8.27 ± 1.61 (19%)	4.78 ± 1.85 (39%)	1.32 ± 2.01 (153%)	27.09 ± 14.00	8.53 ± 1.59 (19%)	52.61 ± 3.54
Cortex all	2.78 ± 1.39 (50%)	1.50 ± 2.36 (158%)	0.51 ± 0.81 (159%)	0.16 ± 0.10 (63%)	8.66 ± 1.59 (18%)	4.55 ± 2.40 (53%)	1.99 ± 2.61 (131%)	23.50 ± 13.91	9.03 ± 1.45 (16%)	53.05 ± 3.06
Hippocampus	3.13 ± 1.34 (43%)	1.82 ± 2.46 (136%)	0.74 ± 1.09 (148%)	0.17 ± 0.09 (54%)	9.84 ± 2.08 (21%)	4.51 ± 2.97 (66%)	2.96 ± 3.87 (131%)	43.45 ± 13.28	10.3 ± 1.98 (19%)	54.34 ± 3.49
Thalamus	4.15 ± 1.54 (37%)	2.37 ± 2.31 (98%)	1.50 ± 1.75 (117%)	0.31 ± 0.21 (68%)	10.87 ± 1.95 (18%)	4.02 ± 3.80 (95%)	3.99 ± 3.42 (86%)	52.70 ± 11.96	11.55 ± 1.75 (15%)	51.91 ± 3.35
Striatum	2.96 ± 1.29 (43%)	0.95 ± 1.30 (136%)	0.36 ± 0.49 (138%)	0.18 ± 0.06 (37%)	10.70 ± 1.86 (17%)	5.25 ± 2.28 (43%)	1.64 ± 1.79 (109%)	38.88 ± 16.19	11.08 ± 1.96 (18%)	55.05 ± 3.66
Hypothalamus	3.62 ± 1.26 (35%)	2.14 ± 2.63 (123%)	0.90 ± 1.11 (124%)	0.21 ± 0.11 (53%)	9.98 ± 1.84 (18%)	4.21 ± 2.78 (66%)	3.29 ± 3.81 (116%)	59.29 ± 11.83	10.55 ± 1.78 (17%)	58.21 ± 5.72
Cerebellum	3.89 ± 1.54 (40%)	1.14 ± 1.51 (133%)	0.24 ± 0.34 (144%)	0.16 ± 0.07 (45%)	9.36 ± 1.41 (15%)	4.96 ± 1.61 (32%)	1.14 ± 0.99 (87%)	31.05 ± 15.55	10.00 ± 1.23 (12%)	55.56 ± 2.96
Brainstem	3.74 ± 1.69 (45%)	1.38 ± 1.73 (125%)	0.45 ± 0.46 (102%)	0.19 ± 0.06 (32%)	11.30 ± 1.69 (15%)	4.65 ± 2.04 (44%)	2.13 ± 1.89 (89%)	34.15 ± 9.07	11.94 ± 1.83 (15%)	53.20 ± 5.90

Figure legends

Figure 1S

Quantification of human amyloid fibrils and human tau in the cortex and hippocampus of 7-month old APP/Tau and ntg rats. LOC (A, C), and Tau13 (B, D) immunoreactive (IR) area in the cortex (A-B) and hippocampus (C-D) of 7-month old ntg (n=11) and APP/Tau rats (n=10). IR area is given in mean percent ± SD. *p< 0.05, **p< 0.01, two-tailed unpaired t-test. E) Representative images of immunofluorescent labeling of whole-brain slices showing LOC (green) and Tau13 (red) antibody labeling in 2 male APP/Tau rats (upper image amyloid- and tau-positive, lower image amyloid-negative, tau-positive). Slices were counterstained with DAPI (blue) to visualize nuclei. Insert in the upper image shows amyloid labeling in the hippocampus. Insert in the lower image shows human tau labeling in the cortex.

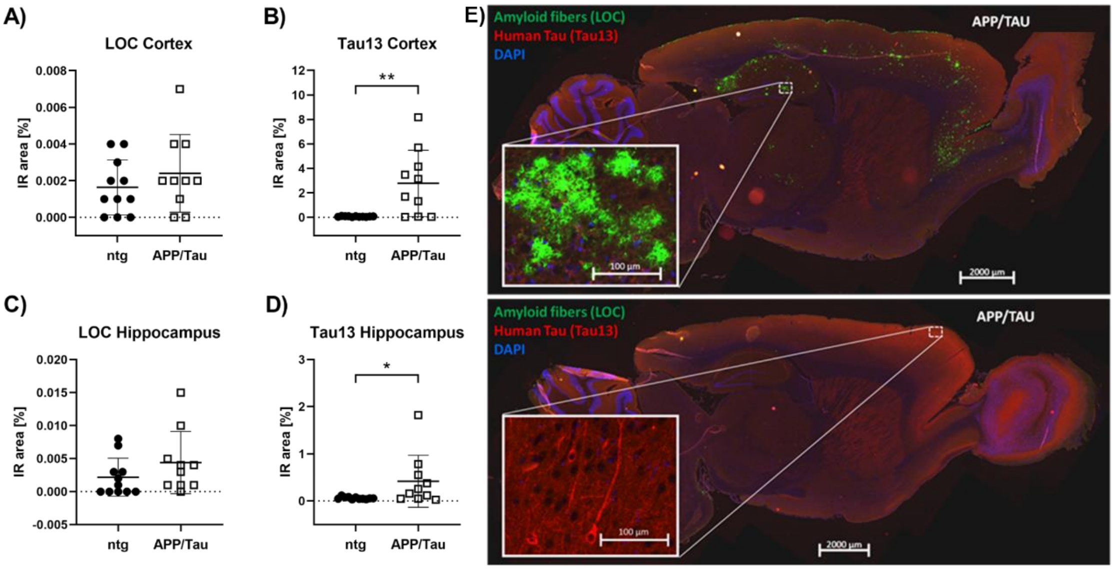
A)
LOC Cortex
IR area [%]
ntg
APP/Tau
B)
Tau13 Cortex
**
C)
LOC Hippocampus
D)
Tau13 Hippocampus
*
E)
Amyloid fibers (LOC)
Human Tau (Tau13)
DAPI
APP/TAU
100 µm
2000 µm

Figure 2S

Quantification of human amyloid fibrils, human tau, astrocytosis, and activated microglia in the cortex and hippocampus of 13-month old APP/Tau and ntg rats. LOC (A, E), Tau13 (B, F), GFAP (C, G), and IBA1 (D, H) immunoreactive (IR) area in the cortex (A-D) and hippocampus (E-H) of 13-month old ntg (n=2) and APP/Tau rats (n=20). IR area is given in mean percent ± SD; two-tailed unpaired t-test yielded no significant differences between ntg and APP/Tau rats.

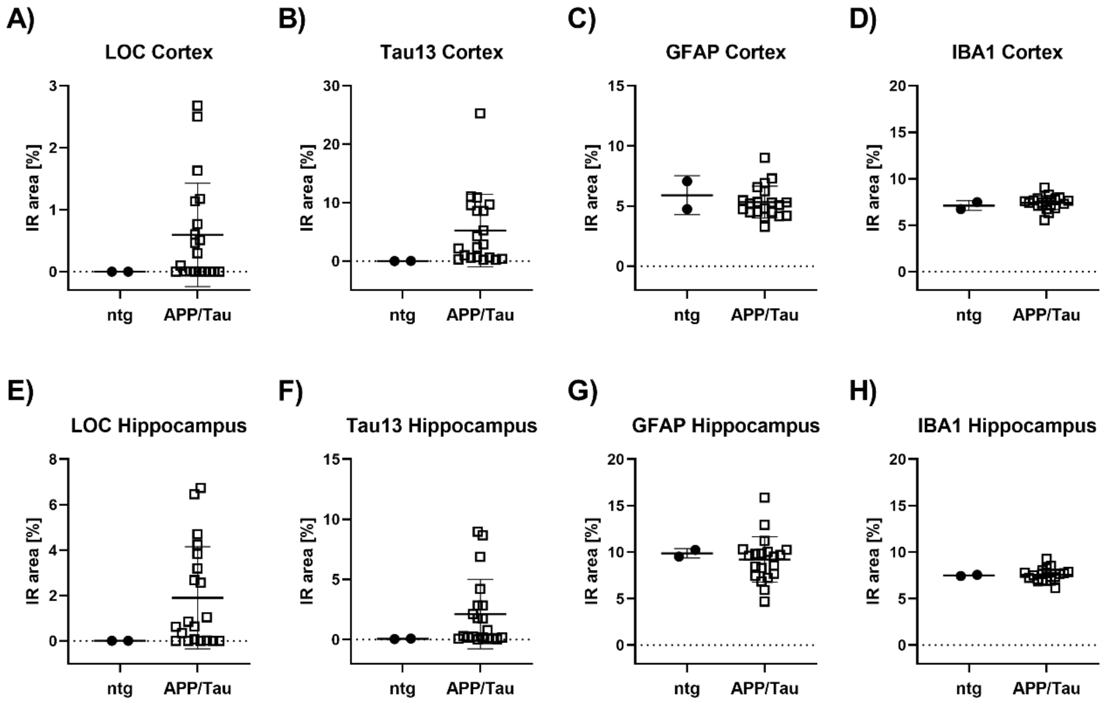

Figure 3S

Quantification of human amyloid fibrils, human tau, astrocytosis, and activated microglia in the cortex and hippocampus of 21-month old APP/Tau and ntg rats. LOC (A, E), Tau13 (B, F), GFAP (C, G), and IBA1 (D, H) immunoreactive (IR) area in the cortex (A-D) and hippocampus (E-H) of 21-month old ntg (n=4) and APP/Tau rats (n=14). IR area is given in mean percent ± SD. *p< 0.05, **p< 0.01, two-tailed unpaired t-test

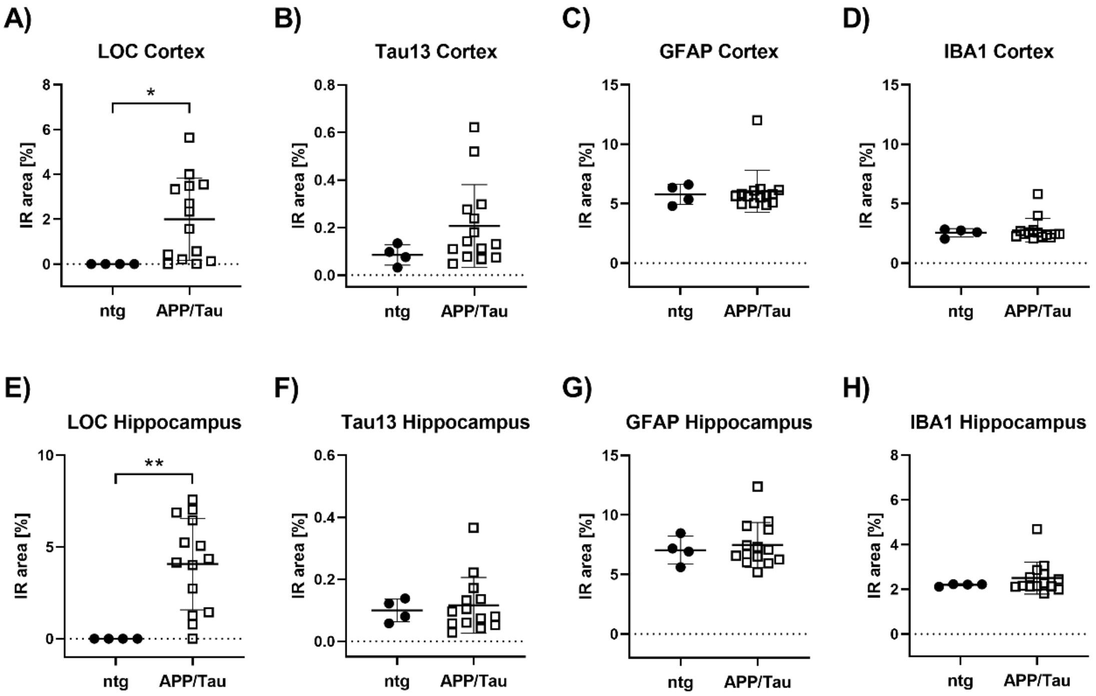

Figure 4S

Correlation of [11C]PiB BP_{ND} obtained with the simplified reference tissue model (SRTM) and the Logan reference tissue (LRT) model. Data points represent calculated BP_{ND}s from all analyzed animals and brain regions. The dotted line equals the line of identity.

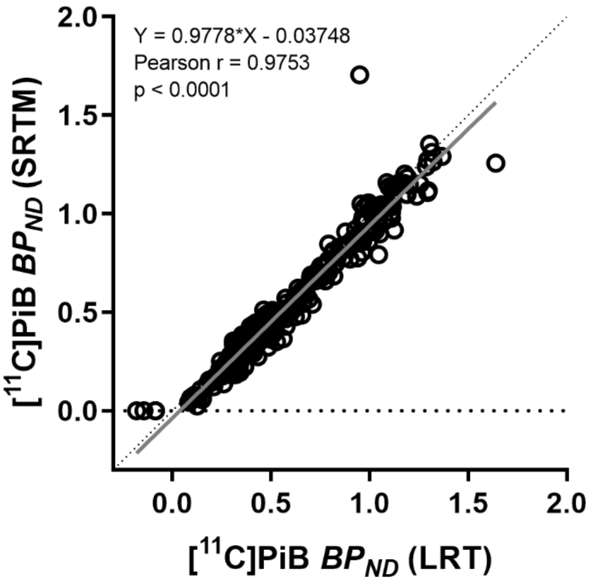

Figure 5S

Correlation of Aβ1-38 (A), Aβ1-40 (B), Aβ1-42 (C), and tau (D) in the CSF with cortical [^{11}C]PiB BP_{ND} obtained with the simplified reference tissue model (SRTM).

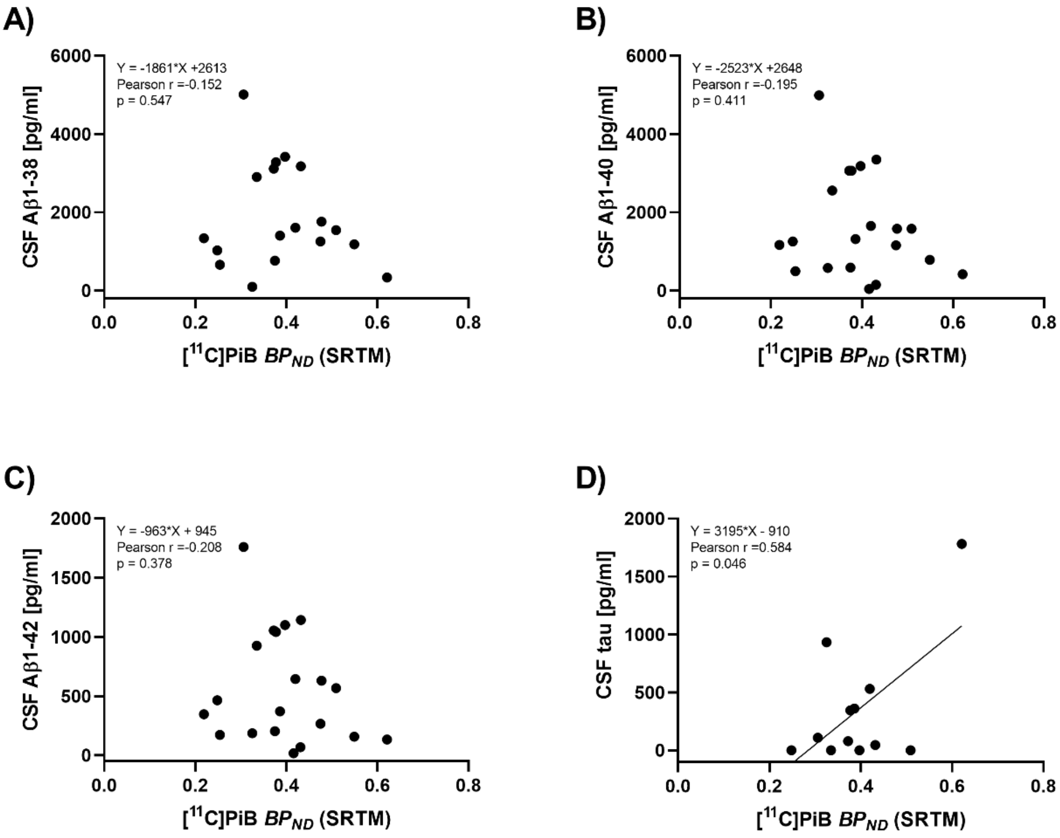

Figure 6S

Representative images of the [^{18}F]THK-5317 compartment modeling procedure extracted from the kinetic modeling tool implemented in PMOD (version 4.002) for the cortex region of one female 21 months old APP/Tau rat. Observed data are shown as green squares, and the model fits as blue lines. Data were modeled using SRTM (yellow curve: TAC from reference region; green squares: TAC from target region) (A), Logan graphical analysis (B), 1T2K model (C), and 2T4K model (D). The yellow curves represent the plasma input function in C and D, whereas the red curves represent the blood input function. The 1T2K model did not provide good fits, whereas the 2T4K model yielded reasonable fits of the PET data.

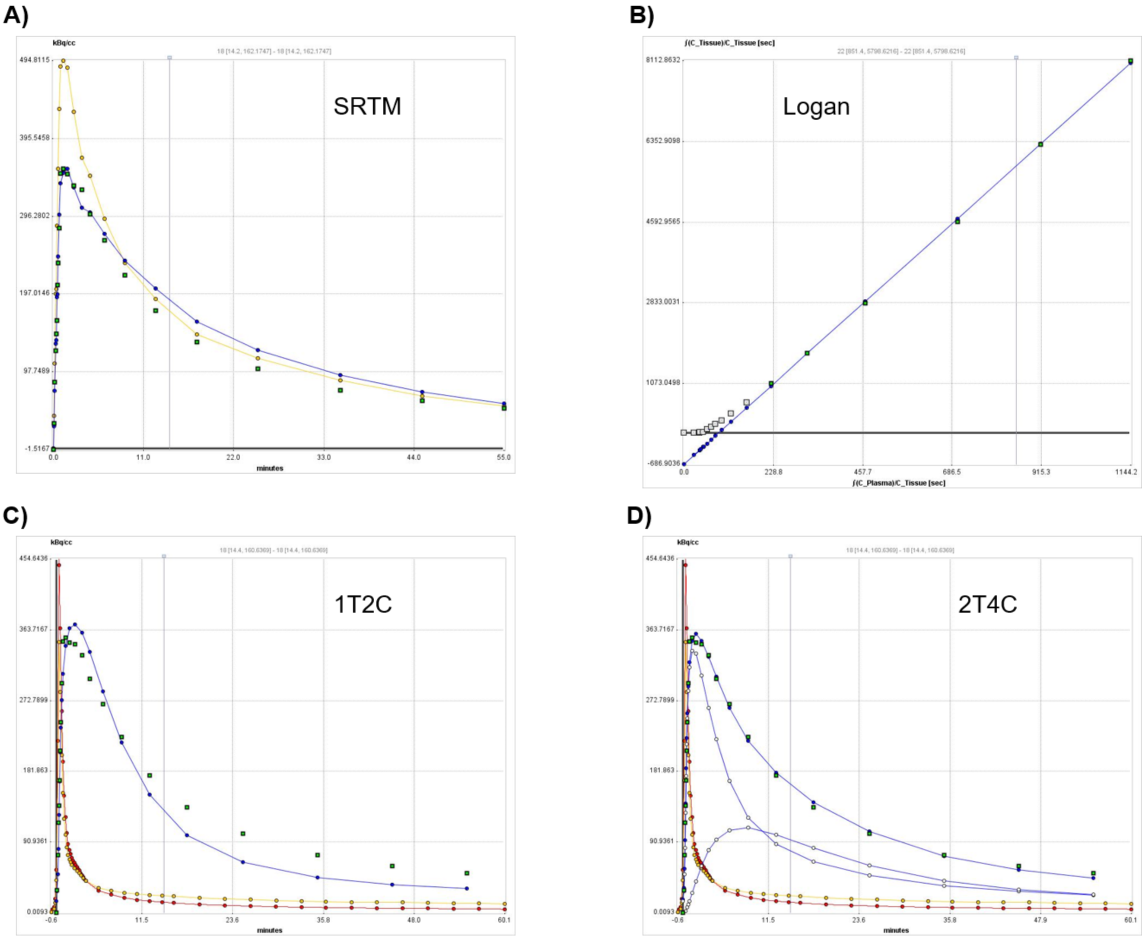
A)
SRTM
kBq/cc
minutes
B)
Logan
∫(C_Tissue)/C_Tissue [sec]
∫(C_Plasma)/C_Tissue [sec]
C)
1T2C
kBq/cc
minutes
D)
2T4C
kBq/cc
minutes

Figure 7S

Correlation of [18F]THK-5317 V_Ts obtained with Logan graphical analysis based on individual and population-based IFs (A). V_Ts calculated in 10 brain regions from 8 rats (80 data points) were used for the correlation. Pearson's correlation coefficient including all rats (r=0.9240; p<0.0001) and excluding 1 rat (labelled with the dashed grey oval; r=0.7316; p<0.0001). Dotted line equals line of identity.

Correlation of [18F]THK-5317 V_Ts obtained with 1T2K (grey circles) and 2T4K (black squares) compartmental model with respective V_Ts obtained with Logan graphical analysis (B). V_Ts calculated in 10 brain regions from 24 rats (21-months old male and female rats, ntg and APP/Tau) were used (240 data points) for the correlation. Pearson's correlation coefficient 1T2K (r=0.9689; p<0.0001) and 2T4K (r=0.9857; p<0.0001). Dotted line equals line of identity.

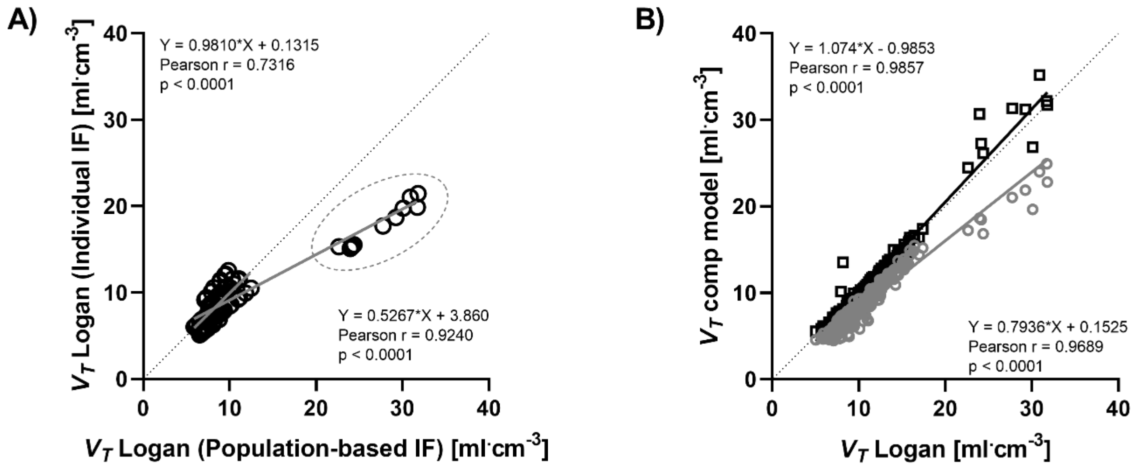

Figure 8S

Correlation of [18F]THK-5317 V_Ts obtained with Logan graphical analysis in the cortex and hippocampus of male and female APP/Tau rats aged 7 and 13 months (female: n=17; male: n=16) (A) and 21 months (female: n=2; male: n=3) (B) with the corresponding tau immunoreactive (IR) area of immunofluorescent labeling (only tau-positive animals are included).

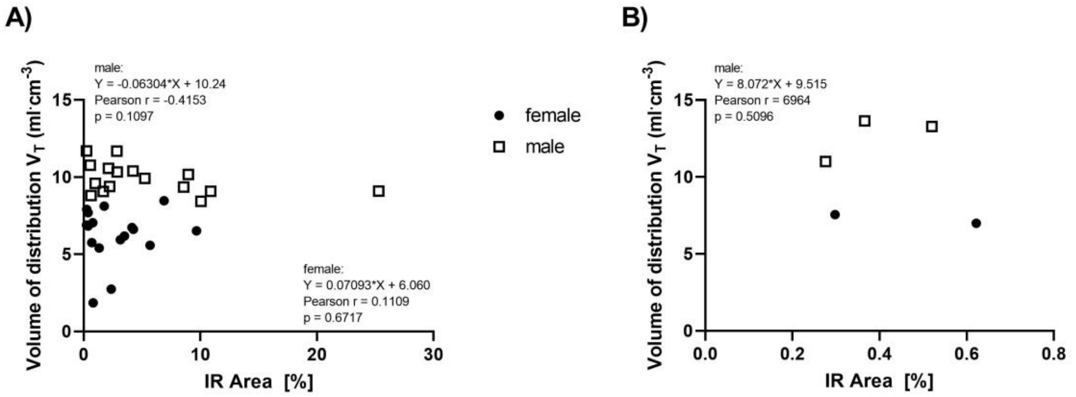

Supplement: Supplementary file 1 — Additional file 1. [file 13195_2021_916_MOESM1_ESM.pdf]
